# Convergent Evolutionary Dead‐End and Breakdown of Hard Chorion in Parental‐Egg‐Care Fish Reproductive Strategies

**DOI:** 10.1111/mec.17816

**Published:** 2025-06-02

**Authors:** Tatsuki Nagasawa, Nagatoshi Machii, Mitsuto Aibara, Mari Kawaguchi, Shigeki Yasumasu, Masato Nikaido

**Affiliations:** ^1^ School of Life Science and Technology Institute of Science Tokyo Meguro‐ku Tokyo Japan; ^2^ Department of Materials and Life Sciences, Faculty of Science and Technology Sophia University Chiyoda‐ku Tokyo Japan

**Keywords:** chorion, evolution, fish, pseudogenes, reproductive strategy

## Abstract

Fish exhibit a diverse array of reproductive strategies adapted to various ecological niches. Parental egg‐care, including live‐bearing, mouth‐brooding, and male egg protection by brood pouches, represents an effective strategy for ensuring larval survival and has emerged independently in multiple lineages. Despite the recognised evolutionary bias that favours a strategy transition from non‐carer to egg‐carer, the genetic mechanisms underlying this bias and the commonalities among parental egg‐care species remain elusive. This study explores the relationship between egg‐care and the chorion hardening system crucial for protecting eggs in non‐care species. By analysing whole genome sequences of 240 species of Acanthopterygii across 25 orders, we discovered that multiple genes associated with chorion hardening have become pseudogenes in various egg‐care species, indicating a collapse of the chorion hardening system in these fish. These findings suggest that the evolutionary bias in fish reproductive strategies not only aims to enhance survival efficiency but also imposes a constraint on egg‐care species, preventing them from reverting to a reproductive strategy relying on a hardened chorion. In particular, alveolin, previously characterised as a single mutant resulting in significantly fragile chorion in medaka, suggests a strong correlation between egg‐care strategy and gene loss. Our results suggest an evolutionary dead‐end because gene loss may impose an evolutionary constraint at the behavioural level. The observed association between gene loss and reproductive strategies provides insights into suitable reproductive environments for each species and may facilitate non‐invasive estimation of reproductive strategies in species with unknown breeding strategies.

## 1 Introduction

Ensuring the survival of offspring by adapting reproductive strategies to the ecological niche is a pivotal factor contributing to the prosperity of the species. Reproductive strategies in organisms vary widely, from mammals that protect a small number of offspring through viviparity to species like some insects that release vast quantities of eggs. The shift between strategies of egg‐guarding and non‐guarding has occurred frequently in the evolution of vertebrates, with over 100 documented instances (Pyron and Burbrink 2014; Morrison et al. 2017). This diversity in reproductive approaches reflects the complex interplay between environmental pressures and evolutionary adaptations aimed at maximising species survival and proliferation.

Fish represent the most diverse group of vertebrates, comprising over half of all extant vertebrate species (Betancur et al. 2017; Nelson et al. 2016). They have successfully adapted to various aquatic environments, covering more than 70% of the Earth's surface, irrespective of factors such as temperature, depth, salinity, or current velocity. The diversity of reproductive strategies in fish is remarkable. Some species, like ocean sunfish and tuna, release their eggs into the water, while others exhibit parental care for their eggs (parental egg‐care). Examples of parental care include live‐bearing in guppies, mouth‐brooding in cichlids, bubble nest protection in Siamese fighting fish and the use of brood pouches by male seahorses (Nelson et al. 2016). Notably, egg‐care strategies have evolved independently in multiple fish lineages, which serve as an example of parallel evolution. For example, mouth‐brooding is observed in arowanas, cichlids and jawfish, which belong to different lineages, and similarly, live‐bearing is found in guppies and black rockfish (Nelson et al. 2016). Despite the prevalence of egg‐care species across diverse lineages, the genetic similarities among them remain largely unexplored.

While reproductive strategies in fish have frequently undergone changes throughout their evolution, it is understood that these changes are not random; rather, they are subject to evolutionary bias. Previous studies, utilising parsimonious inference techniques to reconstruct evolutionary trajectories from the reproductive strategies of existing fish, have consistently revealed that a shift from non‐care to egg‐care strategies is common, whereas a reverse shift is rare (Mank et al. 2005; Balshine and Sloman 2011; Gross and Sargent 1985). However, the specific environmental and genetic factors responsible for this evolutionary bias remain unclear.

To protect embryos from physical stressors such as water flow and bacterial invasion, fish eggs are enveloped in a structure known as the chorion (also referred to as the egg envelope, egg coat, egg membrane or vitelline membrane) (Litscher and Wassarman 2007; Bleil and Wassarman 1980; Smith et al. 2005; Lindsay et al. 2002). After fertilisation, fish chorions undergo a hardening process (Shibata et al. 2012), which endows a single egg with a robust load‐bearing capacity that exceeds 3 kg, as observed in trout (Zotin 1958). However, the chorion's toughness varies among reproductive strategies. For instance, the ovoviviparous platyfish *Xiphophorus maculatus* , belonging to the order Cyprinodontiformes, possesses a chorion that is 30 times thinner and more fragile than that of the oviparous mummichog *Fundulus heteroclitus* , which is from the same order (Kawaguchi et al. 2015). This discrepancy may be attributed to the protective environment within the mother, which may reduce the necessity for a tough chorion. A similar pattern has been observed in other lineages of Syngnathiformes (Kawaguchi et al. 2016), and a study comparing chorion thickness across 55 fish species identified a tendency toward a thinner chorion in egg‐care species (Sano et al. 2017). Nevertheless, conclusive genetic evidence supporting this relationship remains elusive.

**FIGURE 1:**
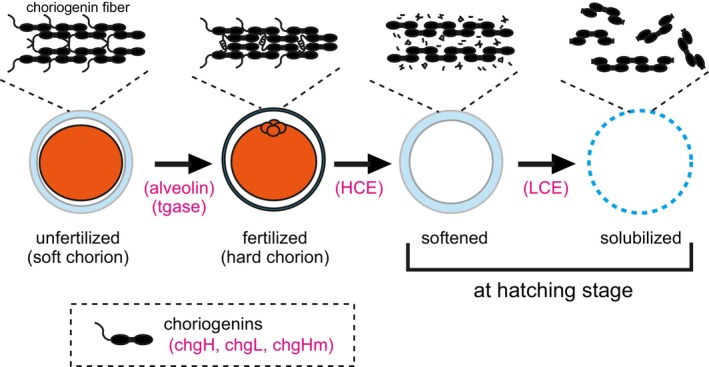
Schematic diagram of the chorion hardening mechanism in medaka, Acanthopterygii. Choriogenins (chgH, chgL and chgHm), the major components of chorion, are synthesised in the liver and transported via the bloodstream to the ovary, and they accumulate around the oocytes to form choriogenin fibres to construct the soft chorion. The cortical alveoli (a kind of cyst distributed on the surface of the oocyte containing secretory granules) of unfertilised eggs contain the astacin metalloprotease, aleveolin, which is secreted by the breakdown of cortical alveoli after fertilisation. The secreted alveolin limitedly proteolyzes the N‐terminus of choriogenin and catalyses the formation of an isopeptide bond between choriogenin molecules by transglutaminase. The formation of this cross‐linked structure completes the formation of hard chorion to protect the embryo during morphogenesis (Shibata et al. 2012). Subsequently, at the hatching stage, the hard chorion is digested by the cooperative action of two hatching enzymes that are secreted from the embryo. First, high choriolytic enzymes (HCE) mainly degrade the N‐terminus of choriogenin, causing the choriogenin fibres to become loose and the chorion to soften. Next, the low choriolytic enzymes (LCE) act on the cleavage site of choriogenin that is exposed by degradation of the N‐terminus of choriogenin, which results in solubilisation of chorion (Yasumasu et al. 2010; Nishio et al. 2024).

The chorion of fish corresponds to the egg membrane in amphibians, the vitelline membrane in birds and reptiles, and the zona pellucida in mammals (Litscher and Wassarman 2015; Jovine et al. 2005). These structures are primarily composed of a glycoprotein, the zona pellucida protein (ZP‐protein) (Litscher and Wassarman 2007; Darie et al. 2004). In fish and other species, the unfertilized egg membrane is formed via the fibrous polymerisation of ZP‐protein during oogenesis in the ovary, with further structural changes occurring post‐fertilisation to complete the formation of the chorion (Lindsay and Hedrick 2004; Shibata et al. 2012; Litscher and Wassarman 2018). The formation of the chorion in fish has been elucidated in studies considering various species, particularly medaka *Oryzias latipes* (Figure 1). In medaka, ZP‐protein is referred to as choriogenin because of its similar molecular behaviour to vitellogenin, the major component of egg yolk; that is, choriogenins and vitellogenins are synthesised in the female liver, transported via the bloodstream, and accumulated in the ovary (Hamazaki et al. 1989; Murata et al. 1997). Choriogenin is further categorised into chgH and chgL, which represent the major components of high and low molecular weights, respectively, along with chgHm (choriogenin H minor), a minor component of chgH, and each is transcribed by distinct coding genes (Murata et al. 1995; Murata et al. 1997; Sugiyama et al. 1998). The formation of hard‐chorion is initiated by the release of alveolin, which accumulates on the surface of the unfertilized egg (cortical alveoli) and is triggered upon fertilisation. This process is completed through the formation of a series of transglutaminase (tgase)‐mediated cross‐linked structures between the choriogenin molecules (Ha and Iuchi 1998; Chang et al. 2002; Yasumasu et al. 2024).

Furthermore, the evolutionary process of the formation of hard‐chorion in fish has been extensively studied. While in amphibians and basal ray‐finned fish, post‐fertilisation structural changes lead to the hardening of the chorion, the common ancestor of teleost fish acquired a tougher chorion through the formation of ZP‐protein cross‐linked structures (Nagasawa et al. 2015). Moreover, subsequent evolutionary processes altered the site of ZP‐protein synthesis from the ovary to the liver, which facilitated the acquisition of a thicker chorion (Sano et al. 2010, 2013, 2022). Subsequently, the duplication of the hatching enzyme gene, which is responsible for the synthesis of chorion‐degrading enzymes, led to the development of a cooperative and efficient digestion system of HCE and LCE (high and low choriolytic enzyme, respectively), thus contributing to further chorion reinforcement (Kawaguchi et al. 2006, 2012; Sano et al. 2014). Therefore, teleosts have expanded their ecological niche by adapting at the reproductive level through successive modifications to their chorion formation and degradation systems.

Recently, an alveolin knockout (KO) medaka has been generated, revealing that the chorion of the alveolin‐deficient individuals possessed only approximately 1/6 of the load‐bearing capacity of that of the wild‐type and exhibited increased permeability. Therefore, the KO medaka formed an insufficient chorion to maintain the internal environment and protect the embryo (Fu et al. 2023). The hardening of the medaka chorion is triggered by the processing of chgH and tgase by alveolin. However, in the alveolin‐KO medaka, these processing events were inhibited. Furthermore, crispant studies of choriogenin genes in medaka have indirectly shown that homozygous mutations in chgH and chgL are lethal, and chgHm‐KO leads to the formation of fragile chorions (Yokokawa et al. 2023). Thus, the mechanism underlying the formation of hard chorion in fish is now well elucidated. Moreover, with the successive publication of high‐quality whole genome sequences of teleosts, it has become feasible to explore the relationship between egg‐care strategies and genes among closely related species. Therefore, we hypothesised that by elucidating the molecular evolutionary processes of genes contributing to fish chorion formation, particularly focusing on egg‐care species, it would be possible to uncover the details of fragile chorion formation and its evolutionary trajectory in these species. In this study, we focused on a cluster of genes associated with the formation of hard chorion in teleost and examined their relationships with egg‐care strategies through a comparative analysis of the genomes of 240 species of Acanthopterygii fish spanning 25 orders, including medaka. Our findings revealed that despite variations in the care strategies across the different lineages, the system responsible for the formation of hard chorion is commonly and irreversibly disrupted, thereby impeding the readaptation to the non‐care strategy.

## 2 Materials and Methods

### 2.1 Isolation of Genes From Genome Sequences and Comparison of Genomic Synteny

The whole genome sequences used in this study were obtained from NCBI‐registered assembled sequence data. The species names and accession numbers of the whole genome sequences used in the analysis are listed in Table S1. Each of the hard‐chorion‐related genes was isolated as described below. First, the genomic locus of each of the genes was estimated by a TBLASTN search using the previously identified amino acid sequence of a closely related species as the query. Furthermore, because the flanking genes were highly conserved, except for HCE, the loci of the flanking genes were estimated in the same method, and the candidate loci of the genes were identified by confirming that they were adjacent to the target genes. Alveolin was flanked by *ttll2* and *igf2r*, alveolin‐like by *wdr45* and *zcchc9*, LCE by *mdga2* and *fkbp3*, chgL and chgHm by *chkb* and *mapk8ip2*, chgH by *tmem263* and *c521/cax2*, and tgase by *nrn1a* and *rreb1*, all of which exhibited high conservation. Based on these features, we confirmed the conservation of genomic synteny by combining the use of web tools such as Genome Data Viewer (Rangwala et al. 2021) and Genomicus (Nguyen et al. 2022) with TBLASTN searches by querying sequences of closely related species. The full‐length sequence of the target genes was isolated by estimating the exon‐intron structure using Genewise (Madeira et al. 2019), using the amino acid sequence of a gene from a closely related species as the query for the sequence of the candidate locus. In particular, to isolate the exon sequences of the N‐terminal region with low conservation, alignments were performed and carefully compared manually with those of closely related species. The target genes were then identified by integrating the results of the molecular phylogenetic tree constructed from the dataset containing the previously identified genes with those of genomic synteny. In several species, some hard‐chorion‐related genes exhibited tandem duplications and HCE genes also exhibited retrocopy, thus forming multicopy genes. However, in this study, the presence or absence of genes was the focus of consideration; therefore, we utilised only a single copy as a representative for analysis.

### 2.2 Construction of Molecular Phylogenetic Tree

Preliminary molecular phylogenetic inference was performed using the sequences of each gene, which revealed a tendency for the gene branches found in the egg‐care species to elongate. Particularly, using the maximum likelihood method, the influence of long‐branch attraction was pronounced even after model optimization (Figure S2C,D), which prompted the use of the neighbour‐joining method for the establishment of the phylogenetic tree. Because of the low conservation of both the N‐ and C‐terminal sequences of astacins (alveolin, alveolin‐like, HCE and LCE), as well as the choriogenins (chgH, chgL and chgHm), the sequences of the protease‐ and ZP‐domains were used for the phylogenetic tree, as described in previous studies (Sano et al. 2010, 2022). Specifically, the amino acid sequences of each gene were aligned using MAFFT (Nakamura et al. 2018), followed by phylogenetic inference using the neighbour‐joining method in MEGA 11 (Tamura et al. 2021), and presented using iTOL (Letunic and Bork 2021). For *tgase*, the molecular phylogenetic and genomic synteny analyses were conducted in a similar method to those of the other genes, that is, the maximum likelihood tree was constructed using full‐length amino acid sequences.

### 2.3 Detection of Pseudogene Fragmentation

Pseudogenized fragments were detected using two methods: visualisation through a VISTA plot (Brudno, Malde, et al. 2003) and comparison with sequences from closely related species using a TBLASTN search. The alveolin gene and chgHm fragments were visualised in the VISTA plot; thus, the VISTA plot results are presented. In this analysis, we utilised genome sequences from species with high genomic sequence quality, which contained both flanking genes on the same scaffold or chromosome, to explore the region between the conserved flanking genes. To compare a wider range of alignments across multiple species using the flanking genes as markers, VISTA analysis was utilised, particularly employing multi‐LAGAN (Limited Area Global Alignment of Nucleotides) (Brudno, Do, et al. 2003). We incorporated knowledge of several key amino acid residues that are crucial for the enzyme activity of the astacin‐family genes, which were elucidated in previous studies (Kawaguchi et al. 2006), such as the consensus sequences of HExxHxxGFxHExxRxDR, SxMHY or cysteine residues for the conformation of higher‐order structures, into our evaluation for sequence conservation. In addition, we assessed the conservation of the intron phases (splice sites) because the astacin‐family genes demonstrated well‐preserved intron phases, except for the retrocopied gene HCE. Furthermore, because of the comparable fragmentation observed in HCE and LCE within the same genus, we only presented results for the representative species. The detection of these pseudogenized fragments and the lineage‐specific comparison of gene losses were conducted based on the phylogenetic relationships referenced from previous studies (Betancur et al. 2017), and fine‐scale phylogenetic relationships were compared according to their respective studies (Stiller et al. 2022; Helmstetter et al. 2016; Hughes et al. 2023). For the statistical analysis of the correlation between parental egg‐care and gene loss, we performed phylogenetic generalised least squares (PGLS) analyses using the *pgls* function in the R package caper (v1.0.3; https://davidorme.r‐universe.dev/caper). Binary data representing the presence or absence of parental egg‐care and gene loss (Table S2) were used for the analysis. The phylogenetic tree was inferred from a neutral genetic region and converted to an ultrametric tree using the R package *ape*; details are provided in the Supplemental Information.

### 2.4 RNA Seq Reanalysis

In this study, we reanalyzed published RNA‐seq data obtained from NCBI repositories. The reference genome assemblies and short read archives (SRAs) were summarized in Table S3. Since genome annotation of *Hippocampus abdominalis* was unavailable, the annotation file was constructed by using Liftoff (Shumate and Salzberg 2021) version 2.35 with default parameters, and the target genes were manually edited. SRA files were retrieved using the SRA Toolkit (Leinonen et al. 2011) (prefetch v3.0.3) and converted to FASTQ format using fasterq‐dump (v3.0.3). Raw reads were compressed using pigz (v2.8) and subjected to quality control processing with fastp (Chen et al. 2018) (v0.20.0) using default parameters. High‐quality reads were then aligned to respective reference genomes using the STAR aligner (Dobin et al. 2013) (v2.7.9a) and quantified via RSEM (Li and Dewey 2011) (rsem‐calculate‐expression v1.3.3). *De novo* assembly of RNA‐seq reads were conducted using Trinity version 2.11.0 (Grabherr et al. 2013). Input FASTQ files underwent identical preprocessing steps as described above prior to assembly. To confirm the absence of target genes, a homology search was conducted using local BLAST. The custom scripts used for this RNA‐seq reanalysis have been deposited to GitHub (https://github.com/MachiiNagatoshi/Nagasawa_et_al_2025/tree/main).

## 3 Results

### 3.1 Identification of Chorion‐Related Genes From Genome Sequences

**FIGURE 2:**
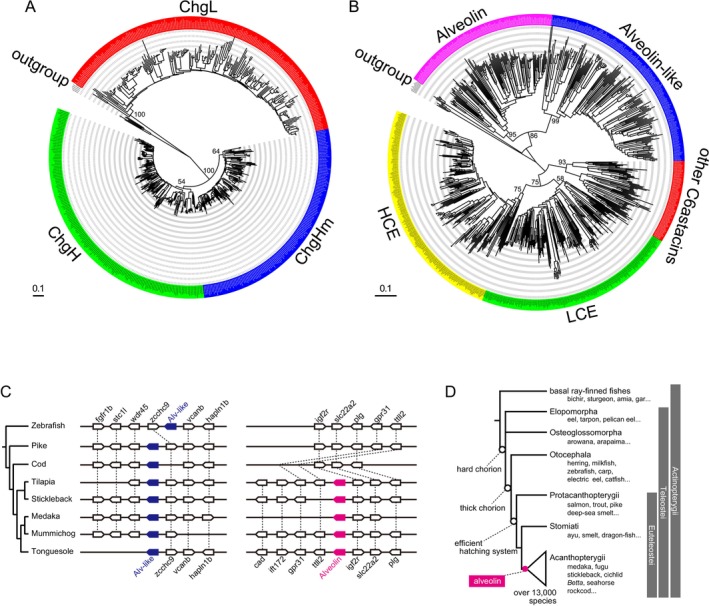
Identification of hard‐chorion‐related genes and the evolution of hard‐chorion. The phylogenetic tree of (A) choriogenins (chgH, chgL and chgHm) and (B) astacin super‐family (alveolin, alveolin‐like, HCE and LCE) genes. Bootstrap values were only attached to nodes that supported the monophyly of each homologous gene (a phylogenetic tree showing all bootstrap values is shown in Figure S2). (C) Genomic synteny of alveolin and alveolin‐like genes. The alveolin gene (magenta pentagon) was only conserved in acanthopterygii, while an alveolin‐like gene (blue pentagon) had already been acquired in non‐acanthomorpha, such as zebrafish, pike, and cod. (D) The stepwise evolutionary process leads to the acquisition of hard‐chorion. During the evolutionary process of fish, the stepwise acquisition of a cross‐link formation system in the teleost common ancestor (Nagasawa et al. 2015), thick chorion by synthesising in the liver of the common ancestor of Otosephala and Euteleostei (Sano et al. 2013), efficient hatching systems in the common ancestor of Euteleostei (Kawaguchi et al. 2012), and alveolin in the common ancestor of Acanthopterygii (used in this study) have led to the completion of hard‐chorion in Acanthopterygii.

First, we isolated the genes associated with the acquisition of hard‐chorion, particularly focusing on Acanthopterygii for reasons discussed later. The list of genes isolated and identified in this study has been compiled into a table along with the previously registered sequences in NCBI (Table S1). Among the choriogenins, chgHm and chgL were adjacent to each other in the genome (between *chkb* and *mapk8ip2*), while chgH was at a different locus (on a different chromosome in most species, between *tmem263* and *c521/cax2*), although chgH and chgHm were similar in their primary sequence and higher‐order structure (Figure S1). While phylogenetic analysis showed low bootstrap support for the monophyly of chgH and chgHm (64% and 54%, Figure 2A,B, Figure S2), genomic synteny analysis provided a robust distinction. The astacin superfamily, including alveolin, HCE and LCE, is particularly diverse in teleost: in addition to new acquisitions such as patristacin, nephrosin and pactacin (Kawaguchi et al. 2021; Nagasawa et al. 2022). HCE genes in particular have undergone frequent translocations (Nagasawa et al. 2019), which required careful comparison for the identification of each gene. Therefore, we isolated these genes through the combination of molecular phylogenetic analysis and genomic synteny, which was similar to our previous comprehensive evolutionary analysis of the entire teleost astacin family (Figure S1) (Nagasawa et al. 2022). As a result, we identified a novel alveolin‐like gene that, despite its sequence similarity to the alveolin identified in medaka, (Figure 2B,C). Interestingly, the alveolin‐like genes were conserved throughout teleosts, including cod, pike, and zebrafish, although alveolin genes were absent in these species. These results indicate that the alveolin gene has been acquired in Acanthopterygii. These new findings indicate that the hard‐chorion formation system that was well understood in medaka studies was established at least since the common ancestor of Acanthopterygii (i.e., after the acquisition of the aleveolin gene; Figure 2D).

### 3.2 Pseudogenization of Hard‐Chorion‐Related Genes in Egg‐Care Species

**FIGURE 3:**
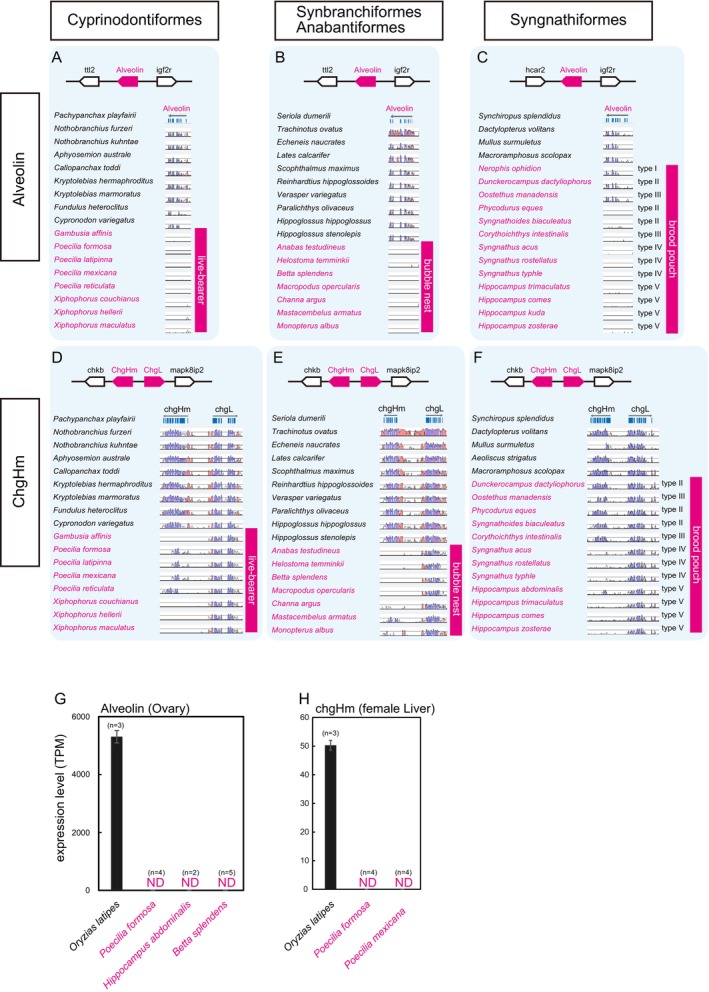
Fragmentation pattern of alveolin and chgHm genes. The pseudogenized fragments of the hard‐chorion‐related genes of Cyprinodontiformes (A and D), Synbranchiformes and Anabantiformes (B and E), and Syngnathiformes (C and F) exhibit clear relationships between the loss of alveolin and chgHm genes and egg‐care strategies (*p* < 0.001: in all analysed lineages). Each panel indicates the degree of fragmentation in the genomic synteny. The results of VISTA alignment for the detection of pseudogenic fragments of alveolins (A–C) and chgHm (D–F) that exhibited conserved genomic synteny are shown using neighbouring genes as indicators. Re‐analysis of RNA‐seq data for (G) alveolin and (H) chgHm. Expression was not detected even by de novo assembly in egg‐care species (magenta).

We compared the gene loss patterns among egg‐care species. In Cyprinodontiformes, all analysed live‐bearing (ovoviviparous) species had lost alveolin, whereas egg‐laying (oviparous) species retained the full‐length sequences of the alveolin (Figure 3A, Figure S3A). A similar pattern was also identified in Anabantiformes and Synbranchiformes (Figure 3B, Figure S3B) which create saliva‐derived bubble nests on the water surface to protect the eggs that they lay inside (Jaroensutasinee and Jaroensutasinee 2001). While in closely related species such as Pleuronectiformes and Carangiformes (including non‐care species), the full‐length sequence of the alveolin gene was conserved; however, the alveolin gene was lost in all analysed species of Anabantiformes and Synbranchiformes (Figure 3B and Figure S3B). In Syngnathiformes, male fish possess a specialised organ for egg‐care known as a brood pouch, which is located on the ventral side of their trunk or tail, and the loss of alveolin was observed only in species with brood pouches (Figure 3C, Figure S3E). It is known that brood pouches are categorised into types I–V, each providing different levels of egg protection, including open types I, II and III and closed types IV and V (Hamilton et al. 2017). The degree of protection increases from types I–V: type I brood pouch is a fully open type; type II is an open type with compartments; type III is an open type that is partially protected by pouch folds; type IV is a closed pouch with bilateral pouch folds; and type V is a sac‐like closed type. All examined species in *Syngnathus* and *Hippocampus*, which possess types IV and V brood pouches, respectively, exhibited alveolin pseudogenes (Figure 3C, Figure S3E). Similar trends were observed in Gobiiformes (mud nests depositors) and Kurtiformes (mouth‐brooders; Figure S3C) and Lophiiformes (covered their eggs with a gelatinous egg veil; Figure S3D). These loss of alveolin genes demonstrated a significant correlation with evolutionary transitions to egg‐care strategies across lineages (*p* < 0.001).

Moreover, in Scorpaeniformes, the genus *Sebastes*, which are live‐bearers, had lost the alveolin gene, while the genera *Cottus* and *Cebidichthys*, which lay eggs onto substrates such as rock and sand and guard them afterwards, retained the full‐length of alveolin gene (Figure S3F). The findings for the species in Syngnathiformes and Scorpaeniformes suggest a correlation between the degree of egg exposure to the external environment and the loss of the alveolin gene. Notably, pseudogenic fragments of alveolin were rarely detected in nearly all species that had undergone the loss of the alveolin gene (Figure 3A–C, Figure S3) suggesting that the alveolin gene may have become a pseudogene during a relatively early evolutionary process in egg‐care species. These findings are consistent with the PGLS analysis results demonstrating a significant correlation between evolutionary transitions to parental egg‐care strategies and alveolin gene loss (*p* < 0.001). On the other hand, newly discovered alveolin‐like genes in this study also exhibited pseudogenization in several egg‐care species, although there was no clear relationship between egg‐care and gene loss as was observed for alveolin (Figure S4).

The loss of chgHm gene was also widespread. In Cyprinodontiformes (Figure 3D), Synbranchiformes, and Anabantiformes (Figure 3E), as well as Syngnathiformes (Figure 3F), some egg‐care species lost chgHm, whereas egg‐laying species retained it. Interestingly, in the order Syngnathiformes, the loss of chgHm appeared to be associated with the degree of protection provided by the brood pouch. Specifically, species with open pouches (types II and III) retained the full length of chgHm, while those with closed pouches (types IV and V) had lost chgHm (Figure 3F). Furthermore, in all of the aforementioned orders, it is expected that the chgHm pseudogenization events occurred more recently than those of alveolin, as evidenced by the pseudogenized fragments of chgHm in some of these species. From the RNAseq, in *Oryzias latipes* (medaka), alveolin (Figure 3G) and chgHm (Figure 3H) were highly expressed in the ovary and female liver, respectively, consistent with previous studies (Murata et al. 1997; Shibata et al. 2012). However, in egg‐care species, neither gene was detected, even with de novo assembly, which supports the loss of alveolin and chgHm.

**FIGURE 4:**
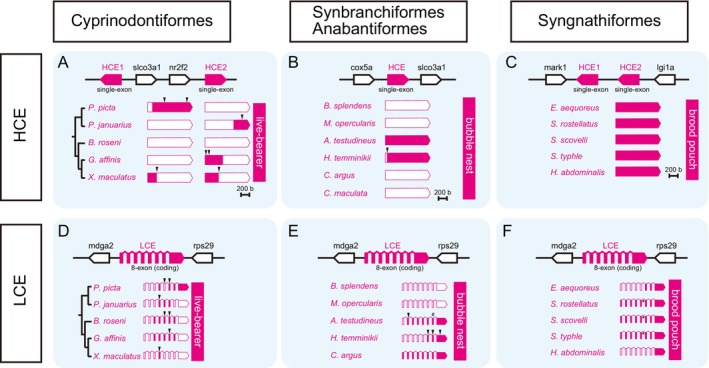
Fragmentation pattern of hatching enzyme genes. The results of the search for pseudogenic fragments of the high choriolytic enzymes (HCE) (A–C) with non‐conservative genomic synteny and low choriolytic enzymes (LCE) (D–F) with short non‐conservative N‐terminal exons are shown as schematic diagrams (A and D: Cyprinodontiformes, B and E: Synbranchiformes and Anabantiformes, C and F: Syngnathiformes).

As shown in our previous study (Nagasawa et al. 2019), although the genomic loci of HCE were not conservative among distantly related species because of retrocopy, the genomic synteny of HCE was conserved among closely related species (Figure S1); therefore, the HCE fragment sequences were searched for regions with conserved genomic synteny (Figure 4A–C). While some egg‐care species exhibited nearly full‐length traces of HCE gene fragments, no such fragments were detected in the genome sequences of other egg‐care species. In Cyprinodontiformes, *Poecilia picta* retained a nearly full‐length but clearly pseudogenized HCE, whereas *Brachyrhaphis roseni* had lost HCE entirely (Figure 4A). Similar trends were observed in Synbranchiformes and Anabantiformes (Figure 4B). In all lineages, the HCE pseudogenization events are suggested to have occurred relatively recently, as evidenced by the presence of almost full‐length pseudogenized HCE gene fragments in species such as *Poecilia picta* and *Helostoma temminkii* . In contrast, in Syngnathiformes, all species retained full‐length HCE genes (Figure 4C). Similarly, in LCE genes, pseudogenization was confirmed in some species of Cyprinodontiformes (Figure 4D), Synbranchiformes, Anabantiformes (Figure 4E), and Syngnathiformes (Figure 4F). The presence of pseudogenized LCE fragments in many species suggests relatively recent pseudogenization. Therefore, the degree of fragmentation of hard‐chorion‐related genes varied from gene to gene, and the alveolin gene in particular was expected to have become a pseudogene at an early stage in the process of shifting to an egg‐care strategy because no traces were observed in any of the analysed species.

### 3.3 Relationship Between Gene Loss and Egg‐Care Strategy

**FIGURE 5:**
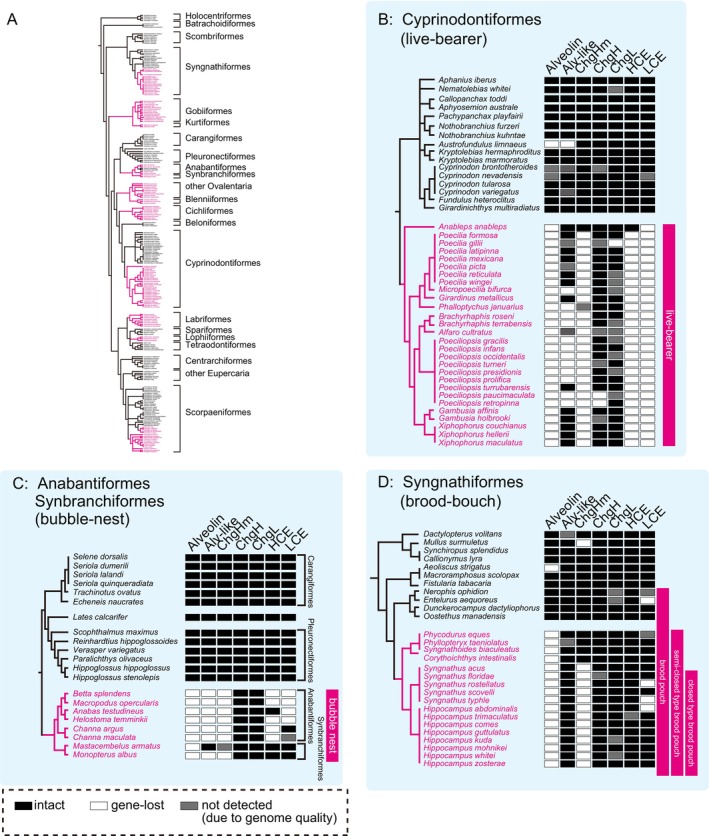
Convergent gene loss of hard‐chorion‐related genes in egg‐care species. (A) Lineage‐independent evolutionary shifts toward egg‐care strategy (shown in magenta) and examples of the egg‐care strategy in Acanthopterygii. Loss of lineage‐independent hard‐chorion‐related genes in egg‐care species: (B) Cyprinodontiformes, (C) Anabatiformes and Synbranchiformes, and (D) Syngnathiformes. The gene losses are indicated as squares (black: intact, grey: not found, white: lost) in the phylogenetic tree of species. The species names of egg‐care species are shown in magenta. The full version of the retaining pattern of hard‐chorion‐related genes of all species analysed in this study is shown in Figure S5.

We comprehensively summarised the relationship between egg‐care strategies and pseudogenization (Figure 5, Figure S5). The phylogeny and parental care traits of Acanthopterygii were referenced from previous studies (Ishimatsu et al. 2018; Helmstetter et al. 2016; Wilson et al. 2001; Harada et al. 2022; Blackburn 2005; Rüder et al. 2006) (Figure 5A).

In Cyprinodontiformes, a clear correlation was observed between pseudogenization and egg care (Figure 5B). All live‐bearers showed alveolin and LCE pseudogenization, and nearly all of the live‐bearers showed chgHm and HCE pseudogenization, with the exception of *Anableps anableps* , which represents an early divergence. In addition, approximately half of the live‐bearers showed pseudogenization of alveolin‐like. Conversely, egg‐laying species retained all hard‐chorion‐related genes, except for *Austrofundulus limnaeus* , which is a unique egg‐laying species known for depositing diapause eggs that endure for several months. A similar pattern was observed in Anabantiformes and Synbranchiformes (Figure 5B). In Syngnathiformes, the pseudogenization pattern showed correlation with brood pouch type: species with closed pouches (types IV–V) lost alveolin and chgHm, whereas species with open pouches also lost alveolin but retained chgHm (Figure 5D). The pseudogenization varied depending on the degree of egg care (Figure 5D). All examined species in *Syngnathus* and *Hippocampus*, which possess types IV and V brood pouches, respectively, exhibited alveolin and chgHm pseudogenes. Conversely, in species retaining open‐type brood pouches, the pseudogenization of only alveolin was confirmed in certain species (Figure 5D). Furthermore, species with typesI–IIII brood pouches retained full‐length chgHm genes, although all Syngnathus and Hippocampus species which possess types IV and V brood pouches, respectively, had lost the chgHm gene (Figure 5D). Consequently, within the Syngnathiformes order, there seems to be a correlation between the level of egg care provided by the brood pouch and the loss of alveolin and chgHm.

These associations between egg‐care and the loss of hard‐chorion‐related genes were particularly evident within the three lineages described above. Moreover, similar correlations were observed across other lineages, including Gobiiformes, Kurtiformes, and Scorpaeniformes (Figure S5). Among egg‐care species, to the best of our knowledge, this study represents the first documented loss of alveolin and chgHm genes, although pseudogenization of the hatching enzymes, HCE and LCE, has been previously reported in platyfish (Kawaguchi et al. 2015), rockfish (Kawaguchi et al. 2008), and seahorse (Kawaguchi et al. 2016). Similarly, the newly discovered alveolin‐like gene was lost in many egg‐care species, although its prevalence was lower than that of alveolin (Figure S5). Interestingly, in species that lay eggs on sandy bottoms or substrates and where parents patrol the surrounding area, such as clownfish, sticklebacks and some cichlids, many genes were retained intact (Figure S5). These results, when considered alongside the findings in Syngnathiformes, may imply a correlation between the degree of egg exposure and gene loss. On the other hand, chgH and chgL, the major components of chorion, were retained in almost all of the analysed species. In the egg‐care species within Cyprinodontiformes, the degradation of other hard‐chorion‐related genes was prominent, whereas tgase, which directly catalyses chorion‐hardening, remained well‐preserved full‐length sequences (Figure S6).

## 4 Discussion

**FIGURE 6:**
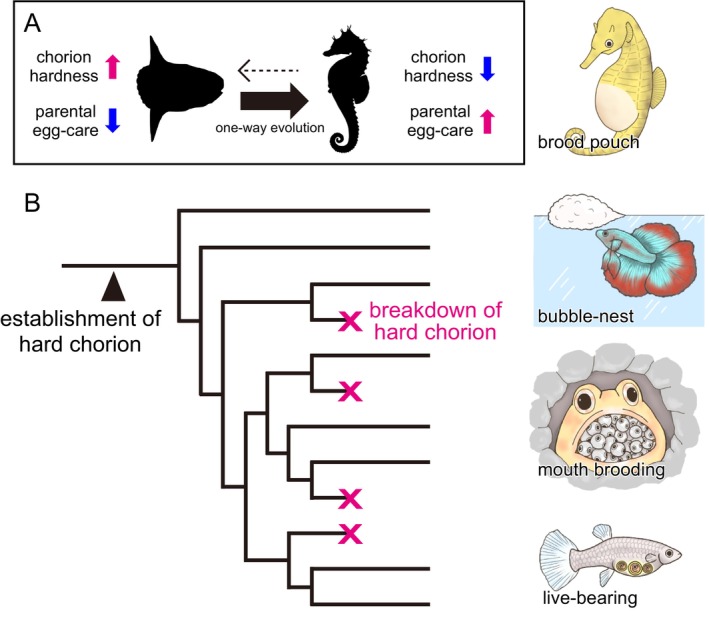
Schematic summary of the convergent evolutionary dead‐end and breakdown of hard‐chorion in egg‐care species. (A) Evolutionary bias in two reproductive strategies of fish. Fish reproductive strategies are characterised by the presence or absence of parental egg care. Non‐egg‐care species adopt a strategy that relies on a hard‐chorion, while egg‐care species choose a strategy that does not depend on a hard‐chorion. The evolutionary shift between these two strategies is unidirectional. (B) The acquisition of hard‐chorion and convergent degradation in egg‐care species was revealed in this study. The hard‐chorion formation system, which was gradually acquired during the evolutionary process of fish, was established in Acanthopterygii through the acquisition of alveolin (indicated by an arrowhead). Subsequently, Acanthopterygii underwent convergent degradation of hard‐chorion with the acquisition of diverse reproductive strategies (marked as X in each branch).

Many fish species are known to adopt parental egg‐care strategies as an adaptation to their environment. Parental egg‐care requires an investment of parental energy, and the choice between various parental egg‐care strategies or opting for no active egg care is recognised as a crucial evolutionary branch point for adaptation to diverse aquatic environments (Gross and Sargent 1985). However, the evolutionary bias in selecting either strategy remains unresolved (Mank et al. 2005; Balshine and Sloman 2011; Gross and Sargent 1985). In this study, we aimed to address this longstanding query by focusing on Acanthopterygii, the largest group within teleost fish. Acanthopterygii has acquired a series of new systems in a step‐by‐step manner over a long evolutionary period to form a hard‐chorion (Figure 2). Furthermore, we found that hard‐chorion‐related genes were commonly and independently lost in many egg‐care species. In other words, the hardening system acquired over a long period during teleost evolution has irreversibly collapsed, thus making it challenging to readapt to a reproductive strategy that relies on a hard‐chorion (Figure 6B). This breakdown of the hard‐chorion formation system was found to occur independently and commonly in multiple lineages, regardless of their egg protection strategy, such as ovoviviparity, nursery in a brooding pouch, bubble nest, or mouth‐brooding. Therefore, parental egg‐care may have resulted in redundancy and the breakdown of the hard‐chorion, thereby promoting a one‐way evolution from non‐care to egg‐care. This study revealed the genetic background underlying the evolutionary biases shaping the reproductive strategies in fish as well as the genetic commonalities across the diverse egg‐care strategies. It is possible that the irreversibility of reproductive strategies in fish is not solely the result of strong evolutionary pressure that favours high‐fitness strategies but rather the consequence of the collapse of the hard‐chorion system because of the long‐term evolutionary persistence of egg‐care.

Vertebrates have independently acquired parental care strategies over 100 times across different lineages (Pyron and Burbrink 2014; Morrison et al. 2017), and similar to fish, this adaptation is irreversible (Reynolds et al. 2002). The relationship between the irreversible adaptation to egg‐care strategies in Acanthopterygii, as elucidated in this study, and gene loss is believed to have substantial implications for the understanding of adaptation to parental care across all vertebrate species. For instance, mammals, which are representative of viviparity, may have also encountered an evolutionary dead end because of irreversible gene loss. They have lost vitellogenin, an egg yolk protein, making it challenging to readapt to a development style that is dependent on egg yolk nutrition (Brawand et al. 2008). Furthermore, our previous study showed that hatching enzyme genes that are conserved in non‐mammalian vertebrates are lost in Mammalia (Nagasawa et al. 2022). Thus, it is possible that irreversible genetic transitions, such as gene loss, may provide evolutionary direction even in non‐teleost species. In recent years, the decoding of whole genome sequences of a vast number of species has allowed for detailed genomic comparisons based on reproductive strategies, such as those detailed in this study. This comparative analysis may lead to elucidating the molecular mechanisms underlying the evolutionary constraints on reproductive patterns across all vertebrates.

Among the hard‐chorion‐related genes examined in this study, the loss of the alveolin gene, in particular, was observed in the largest number of egg‐care species (Figure S5). Although this study is the first report of fish lacking the alveolin gene, it is notable to observe its frequent absence across numerous egg‐care species. Furthermore, these findings are consistent with a previous report (Fu et al. 2023), which revealed that although alveolin‐KO medaka formed a fragile chorion, they could be successfully raised through gentle handling during the early developmental stages. Based on these findings, it is expected that the loss of alveolin is a major factor that leads to the irreversible egg‐care strategy. However, it remains controversial whether chorion hardening is completely inhibited in egg‐care species. According to the study by Hu et al. (2023), in alveolin‐KO medaka, the complete inhibition of chorion hardening does not occur; instead, it has been reported that hardening begins with a delay in timing. Our analysis revealed the maintenance of tgase, which catalyses the final hardening reaction of the egg envelope (Ha and Iuchi 1998; Chang et al. 2002; Yasumasu et al. 2024), in egg‐care species (Figure S6). Therefore, careful analysis of the function of tgase in egg‐care species is considered necessary. The newly discovered alveolin‐like genes, most resembling the alveolin gene within the astacin superfamily, did not exhibit as strong a correlation with egg‐care as that of the loss of the alveolin gene. However, the alveolin‐like genes were lost in some egg‐care species, which may imply a function for the alveolin‐like gene found for the first time in this study (Figures S4 and S5). Combined with the observation of the disruption of the chgHm gene in various egg‐care species, it is anticipated that future comparative analyses will require the generation of double or triple mutants of alveolin, alveolin‐like, and chgHm genes, in comparison with the chorion of non‐care species.

In this study, we revealed the patterns of evolution and loss of the chorion‐related genes in Acanthopterygii through a comprehensive comparison of genome sequences. Previously, it was suggested that there might be differences in the egg envelope hardening system between species, such as arowana, eel and zebrafish, which synthesise egg envelope proteins in the ovaries, and Acanthopterygii species, which synthesise these proteins in the liver (Sano et al. 2022). The establishment of the alveolin gene as the trigger for the hardening of the egg envelope in the common ancestor of Acanthopterygii, as demonstrated in this study, may serve as molecular evidence indicating the differences in the hardening systems at the molecular level. We have previously demonstrated that the egg envelope hardening system in fish has undergone gradual changes during evolution (Kawaguchi et al. 2006; Nagasawa et al. 2015; Sano et al. 2022). However, because all of the analysed hard‐chorion‐related genes (except those in egg‐care species) were well conserved in Acanthopterygii, this suggests that the mechanism for chorion hardening may have become stable throughout the Acanthopterygii lineage.

In previous studies, a double‐KO medaka for chgH/chgHm has been generated; however, the characteristics of the chorion in a single‐KO medaka for chgHm have not been elucidated (Yokokawa et al. 2023). This previous study indicated that the double‐KO of chgH/chgHm produces more friable chorions compared to a single‐KO of chgH, which indicates the importance of chgHm in the formation of hard chorion. However, the role of chgHm remains unclear. In this study, we found that many egg‐care species have lost ChgHm (Figure S5). These findings are consistent with a previous study (Kawaguchi et al. 2015), which revealed that platyfish, a live‐bearing species, lost chgHm and also implied the presence of a relationship between chgHm and egg care. However, chgH and chgL were well conserved in most species, regardless of the presence or absence of egg‐care behaviour (Figure S1). These findings are consistent with the observation that chgH‐ and chgL‐KO medaka exhibited lethality, likely because of the inability to fully prevent polyspermy (Murata and Kinoshita 2022; Yokokawa et al. 2023). In addition, it is possible that in egg‐care fish, the essential minimum chorion formation system required for development may not be lost. On the other hand, egg‐care species tend to exhibit a lower expression level of chgH and chgL (Kawaguchi et al. 2015). In medaka, the transcription of chgH and chgL requires the presence of the female oestrogen hormone (E3), and it is known that males exhibit enhanced transcription of chgH and chgL when exposed to E3 (Murata et al. 1997). Similarly, several genes are known to undergo substantial expression level changes in the ovaries when exposed to E3 (Sun et al. 2011; Dong et al. 2023; Wang et al. 2023), and it is possible that egg‐care species may share other genes that have consistently lower expression levels. In the future, performing comparative analyses of gene expression levels between egg‐care species, considering their exposure to E3, and their closely related egg‐care and non‐care species, may reveal novel genes that are involved in robust egg envelope formation.

The loss of hard‐chorion‐related genes in egg‐care species, as found in this study, may serve as a supporting factor in estimating reproductive strategies. Given the irreversibility of the shift toward egg‐care, environmental changes that render parental care impossible could significantly reduce fitness. Therefore, distinguishing between egg‐care and non‐care strategies is an important indicator from a conservation perspective. The methodology used in this study allows for non‐invasive estimation of reproductive strategies based on DNA extracted from whole genome sequences or tissues, such as fin clips. This approach may be particularly useful for endangered species or fish with inaccessible breeding grounds, such as deep‐sea fish. While the reproductive strategies of many fish species remain unclear—for example, whether the climbing perch ( *Anabas testudineus* ) practices egg‐care (Zworykin 2020)—our results showed that *A. testudineus* exhibits a gene loss pattern similar to that of the closely related bubble‐nesting species *Betta splendens* (Figure 5C). These results suggest that *A. testudineus* could be an egg‐care species, although further validation is necessary. However, this genetic approach should not be regarded as a definitive indicator in all cases. For instance, African cichlids (mouth‐brooder), exhibit little or no gene loss, even in the egg‐care species. This retention of genes may be due to the relatively recent evolution of mouth‐brooding traits (approximately 10 million years ago) (Genner et al. 2007), suggesting that pseudogenization at the genomic level has not yet occurred. In such cases, the relationship between egg‐care and gene loss may not be evident. To improve the reliability of this method, incorporating additional information, such as transcriptional regulation and gene expression levels of hard‐chorion‐related genes, could enhance its potential as a complementary tool for estimating reproductive strategies in future studies.

## Author Contributions

This study was conceptualised and analysed mainly by Tatsuki Nagasawa. The RNA‐seq analysis using public data was performed by Tatsuki Nagasawa and Nagatoshi Machii. A literature review of fish reproductive strategies was performed by Tatsuki Nagasawa, Mitsuto Aibara and Mari Kawaguchi. Interpretation and discussion of the analysis results, as well as the preparation and revision of the manuscript, were conducted collaboratively by all authors.

## Conflicts of Interest

The authors declare no conflicts of interest.

## Supporting information


Data S1.

**Table S1.** List of all species, gene sequences and accession numbers used in this study.

**Table S2.** Binary data of gene lost and egg‐care for PGLS analysis.

**Table S3.** List of accession number of RNA‐seq data and genome assembly.

## Data Availability

This study did not involve the collection or use of any biological samples. All data used in this research—comprising publicly available gene sequences, whole genome sequences, and transcriptomic datasets—were obtained from established public databases. Detailed sources, accession numbers of all datasets, and all analysis scripts used in this study are provided in the Supplemental Information. Therefore, this research is fully compliant with the Nagoya Protocol (https://www.cbd.int/abs/) on Access and Benefit‐Sharing, as no genetic resources subject to access and benefit‐sharing obligations were utilised.
