# Supplementary material for: Convergent Evolutionary Dead‐End and Breakdown of Hard Chorion in Parental‐Egg‐Care Fish Reproductive Strategies
Source: Mol Ecol. 2025 Jun 2;34(13):e17816. doi: 10.1111/mec.17816 (PMC12186722; doi:10.1111/mec.17816)
Supplement: Supplementary file 1 — Data S1. [file MEC-34-e17816-s003.pdf]

MOLECULAR ECOLOGY

Supplemental Information for:

Convergent evolutionary deadend and breakdown of hard chorion in parental-egg-care fish reproductive strategies

Tatsuki Nagasawa, Nagatoshi Machii, Mitsuto Aibara, Mari Kawaguchi, Shigeki Yasumasu, Masato Nikaido

Table of Contents:

R scripts for statistical analysis	Page 2-3
Detailed results of PGLS analyses	Page 3-5
Supplemental figures	Page 6-20
Supplementary Table list	Page 21
Dataset used for PGLS analysis	Page 21-30
Reference	Page 30

MOLECULAR ECOLOGY

Supporting information

An example of statistical analysis method

The statistical analysis of gene loss in parental-egg-care species (PGLS) was basically conducted following the example R code provided below. The a-tubulin sequences used for phylogenetic tree reconstruction and the resulting tree in Newick format are included at the end of this document. Since the inferred tree based on α-tubulin sequences was largely consistent with the species phylogeny shown in Fig. 5A, we used this tree for the analysis. Prior to the analysis, the tree was converted to an ultrametric tree using the chronos function in the R package ape.

```
setwd("C:/alveolin/alv/alv2/")
library(ape)
library(phytools)
library(caper)
library(TreeTools)


test_tree <- read.tree("250527Cyprinodontiformes_tub.nwk")

test_data <- read.csv("250506Cyprinodontiformes_Alv_data.csv")
test_data <- test_data[, !colnames(test_data) %in% c("X", "X.1")]

test_data$alv[test_data$guarding == 1 & test_data$alv == 1][1] <- 0


test_tree_rooted <- RootTree(test_tree, outgroupTips="Pacpla")

test_tree_rooted$edge.length[test_tree_rooted$edge.length == 0] <- 1e-6

is.ultrametric(test_tree_rooted)

test_tree_ultra <- force.ultrametric(test_tree_rooted, method="nnls")

is.ultrametric(test_tree_ultra)
comp_data <- comparative.data(test_tree_ultra, test_data, "species_name",
warn.dropped=TRUE)

test_tree_ultra$node.label <- NULL
comp_data <- comparative.data(test_tree_ultra, test_data, "species_name",
warn.dropped=TRUE)
```

MOLECULAR ECOLOGY

```
pgls_model <- pgls(alv ~ guarding, data=comp_data, lambda="ML")

summary(pgls_model)
```

PGLS results in Cyprinodontiformes Alveolin

```
Call:
pgls(formula = alv ~ guarding, data = comp_data, lambda = "ML")

Residuals:
    Min      1Q  Median      3Q     Max 
-2.6073  0.1013  0.1280  0.3135  0.4813 

Branch length transformations:

kappa  [Fix]  : 1.000
lambda [ ML]  : 1.000
   lower bound : 0.000, p = 3.7863e-12
   upper bound : 1.000, p = 1         
   95.0% CI   : (1.000, NA)
delta  [Fix]  : 1.000

Coefficients:
               Estimate  Std. Error   t value Pr(>|t|)    
(Intercept) -0.14749679  0.13694377   -1.0771   0.2906    
guarding     0.99999629  0.00090292 1107.5117   <2e-16 ***
---
Signif. codes:  0 ‘***’ 0.001 ‘**’ 0.01 ‘*’ 0.05 ‘.’ 0.1 ‘ ’ 1

Residual standard error: 0.6994 on 28 degrees of freedom
Multiple R-squared:     1,	Adjusted R-squared:     1 
F-statistic: 1.227e+06 on 1 and 28 DF,  p-value: < 2.2e-16
```

PGLS results in Cyprinodontiformes ChgHm

```
Call:
pgls(formula = chghm ~ guarding, data = comp_data, lambda = "ML")

Residuals:
     Min       1Q   Median       3Q      Max 
-282.176   -1.372    0.199    1.041  294.425 

Branch length transformations:

kappa  [Fix]  : 1.000
lambda [ ML]  : 1.000
   lower bound : 0.000, p = 2.7866e-11
   upper bound : 1.000, p = 1
```

```
   95.0% CI    : (1.000, NA)
delta  [Fix]  : 1.000

Coefficients:
               Estimate  Std. Error   t value Pr(>|t|)
(Intercept) -0.74170444 15.09366005   -0.0491   0.9612
guarding     0.99998739  0.00099518 1004.8290   <2e-16 ***
---
Signif. codes:  0 ‘***’ 0.001 ‘**’ 0.01 ‘*’ 0.05 ‘.’ 0.1 ‘ ’ 1

Residual standard error: 77.09 on 28 degrees of freedom
Multiple R-squared:     1,     Adjusted R-squared:     1
F-statistic: 1.01e+06 on 1 and 28 DF,  p-value: < 2.2e-16
```

PGLS results in Anabantiformes & Synbranchiformes Alveolin

```
Call:
pgls(formula = alv ~ guarding, data = comp_data, lambda = "ML")

Residuals:
    Min      1Q  Median      3Q     Max
-2.7312  0.0000  0.1821  0.1821  0.1821

Branch length transformations:

kappa  [Fix]  : 1.000
lambda [ ML]  : 0.000
   lower bound : 0.000, p = 1
   upper bound : 1.000, p = < 2.22e-16
   95.0% CI    : (NA, 0.263)
delta  [Fix]  : 1.000

Coefficients:
            Estimate Std. Error t value  Pr(>|t|)
(Intercept) 0.000000   0.072984   0.000         1
guarding    0.937500   0.089387  10.488 5.037e-10 ***
---
Signif. codes:  0 ‘***’ 0.001 ‘**’ 0.01 ‘*’ 0.05 ‘.’ 0.1 ‘ ’ 1

Residual standard error: 0.6014 on 22 degrees of freedom
Multiple R-squared: 0.8333,    Adjusted R-squared: 0.8258
F-statistic:   110 on 1 and 22 DF,  p-value: 5.037e-10
```

PGLS results in Syngnathiformes Alveolin

```
Call:
pgls(formula = alv ~ guarding, data = comp_data, lambda = "ML")

Residuals:
    Min      1Q  Median      3Q     Max
-1.5866  0.0000  0.0000  0.4533  0.4533
```

```
Branch length transformations:

kappa  [Fix]  : 1.000
lambda [ ML]  : 0.000
   lower bound : 0.000, p = 1
   upper bound : 1.000, p = 1
   95.0% CI   : (NA, NA)
delta  [Fix]  : 1.000

Coefficients:
              Estimate Std. Error t value  Pr(>|t|)
(Intercept) 8.6972e-08 6.8657e-02  0.0000         1
guarding    7.7778e-01 1.1212e-01  6.9372 5.776e-07 ***
---
Signif. codes:  0 ‘***’ 0.001 ‘**’ 0.01 ‘*’ 0.05 ‘.’ 0.1 ‘ ’ 1

Residual standard error: 0.5424 on 22 degrees of freedom
Multiple R-squared: 0.6863,   Adjusted R-squared: 0.672
F-statistic: 48.12 on 1 and 22 DF,  p-value: 5.776e-07
```

PGLS results in Lophiiformes Alveolin

```
Call:
pgls(formula = alv ~ guarding, data = comp_data, lambda = "ML")

Residuals:
    Min      1Q  Median      3Q     Max
-2.4046  0.0000  0.3435  0.3435  0.3435

Branch length transformations:

kappa  [Fix]  : 1.000
lambda [ ML]  : 0.000
   lower bound : 0.000, p = 1
   upper bound : 1.000, p = 0.00041464
   95.0% CI   : (NA, 0.522)
delta  [Fix]  : 1.000

Coefficients:
              Estimate Std. Error t value Pr(>|t|)
(Intercept) 1.6086e-07 1.8002e-01  0.0000 0.999999
guarding    8.7500e-01 2.1109e-01  4.1451 0.002503 **
---
Signif. codes:  0 ‘***’ 0.001 ‘**’ 0.01 ‘*’ 0.05 ‘.’ 0.1 ‘ ’ 1

Residual standard error: 0.8569 on 9 degrees of freedom
Multiple R-squared: 0.6562,   Adjusted R-squared: 0.6181
F-statistic: 17.18 on 1 and 9 DF,  p-value: 0.002503
```

MOLECULAR ECOLOGY

Figure S1

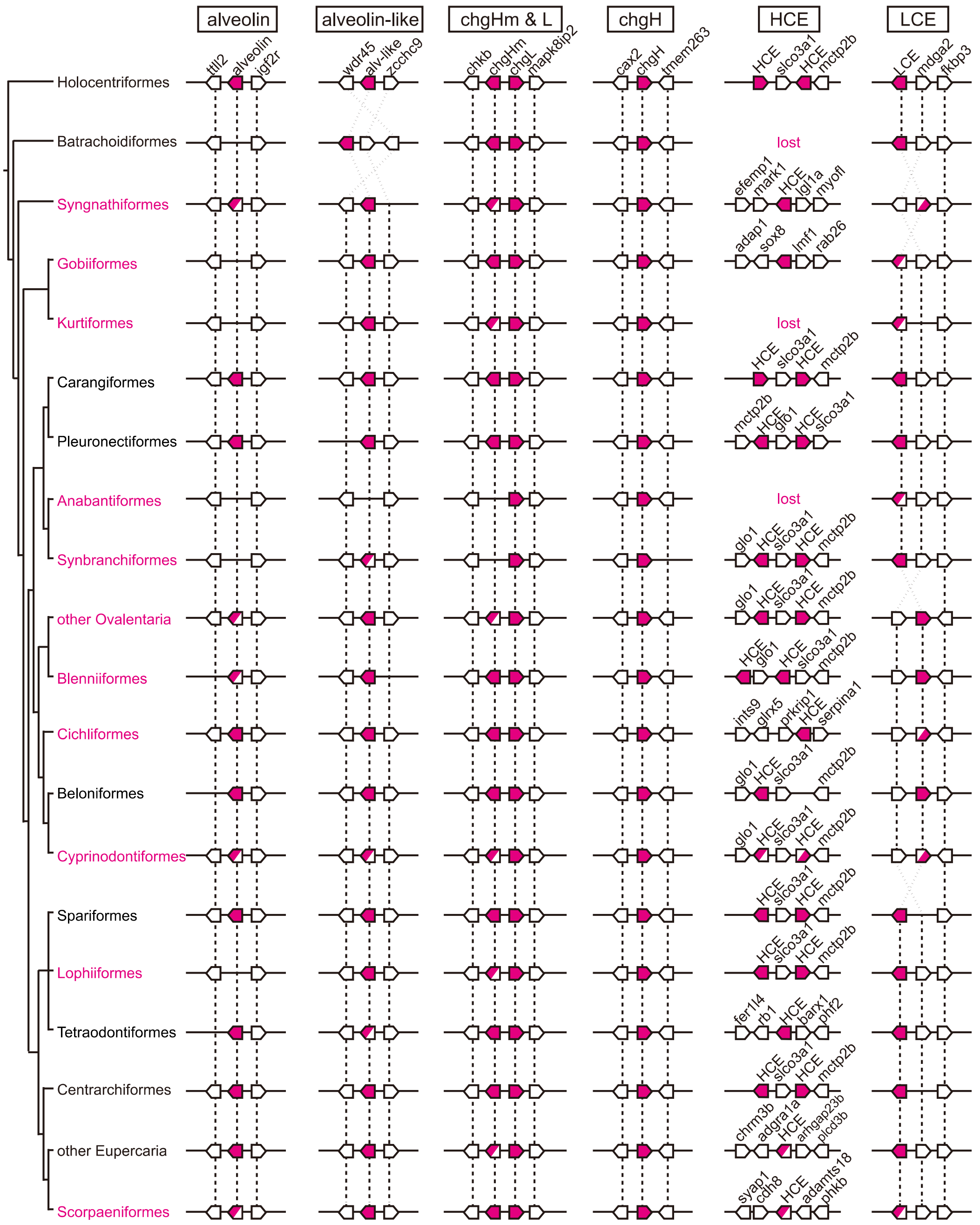

MOLECULAR ECOLOGY

Fig. S1 Genomic synteny of hard-chorion–related genes
The schematic illustrations of the genomic synteny of hard-chorion–related genes in the representative species of each order. The phylogenetic relationships of each order are shown on the left, and the genomic synteny of the hard-chorion–related genes identified in this study is presented on the right. Orders containing species with egg-guarding are highlighted in magenta. The presence or absence of genes is indicated by pentagons along with information on the transcriptional direction, with homologous genes connected by dashed lines. Particularly, the hard-chorion–related genes are indicated in magenta. Orders of all the analyzed species that have lost the gene are represented by no-pentagons, while orders with a mixture of gene presence and absence patterns within the same order are represented by half-filled pentagons in magenta. Because the genomic positions of the high choriolytic enzyme (HCE) genes vary across lineages, neighboring genes were individually listed for each lineage. In lineages within the orders of all species that have lost the HCE, there are no neighboring genes for comparison; therefore, they are marked as 'lost'. In all analyses, several species exhibited tandem duplication of multi-copied genes, which was compressed to a single copy in our analyses. In several lineages, the ancestral HCE gene of the retrocopy was retained, but this information has been omitted.

MOLECULAR ECOLOGY

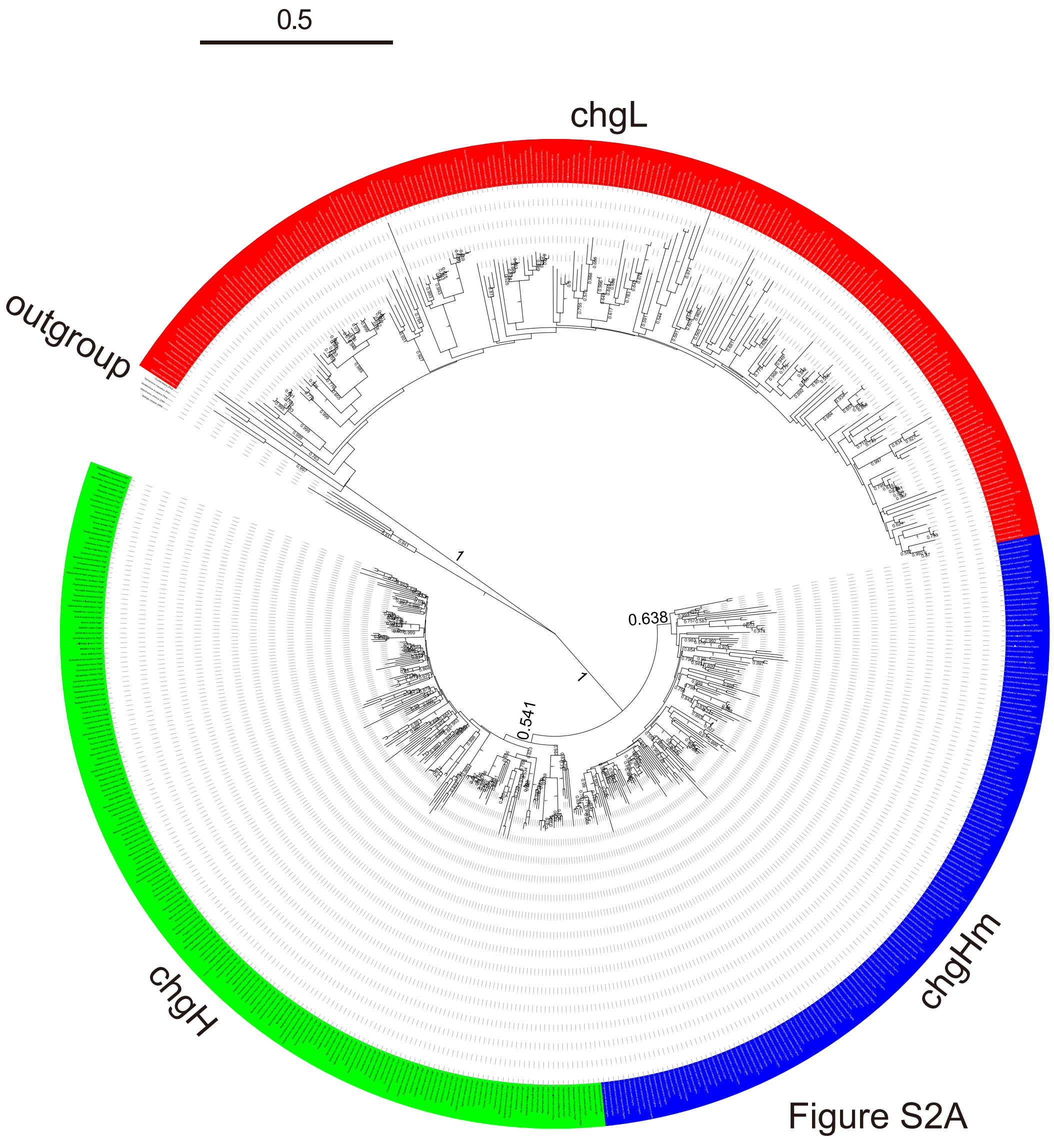

Figure S2A

MOLECULAR ECOLOGY

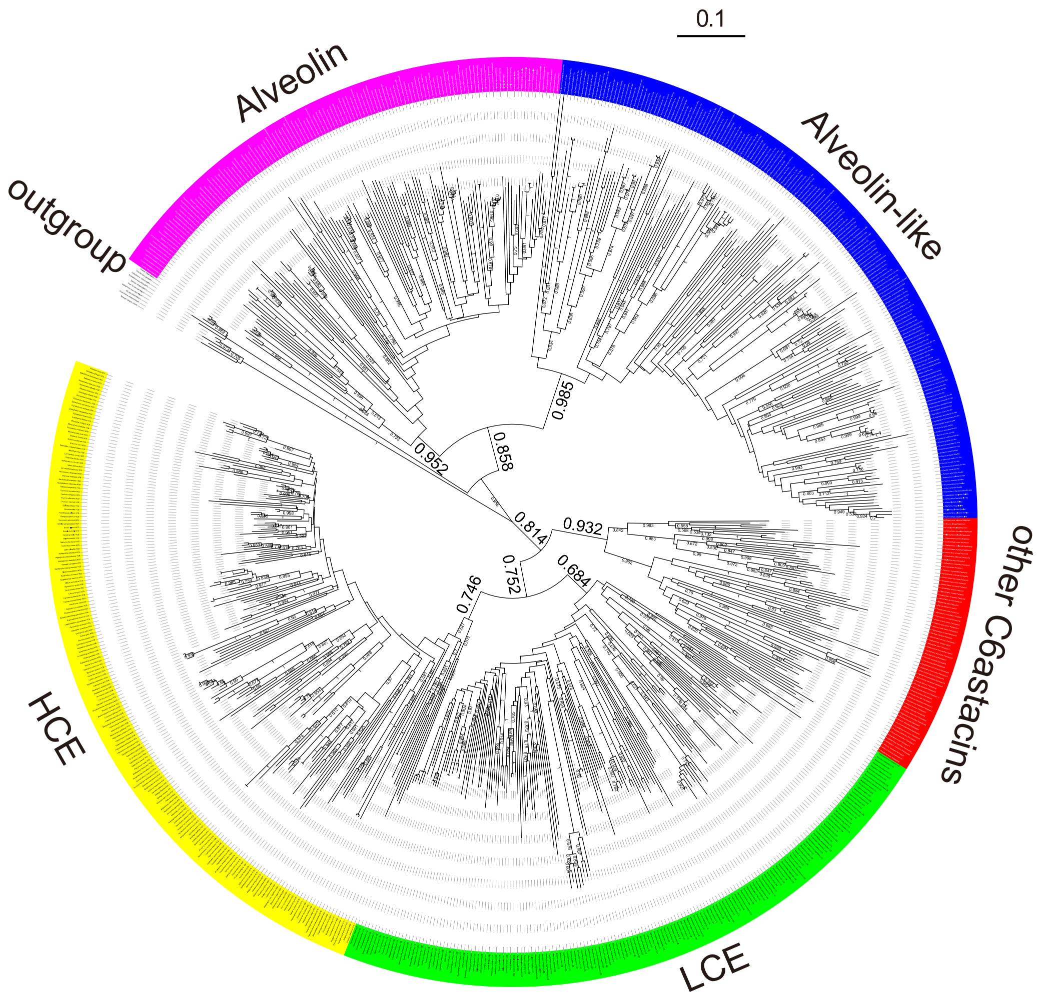

Figure S2B

MOLECULAR ECOLOGY

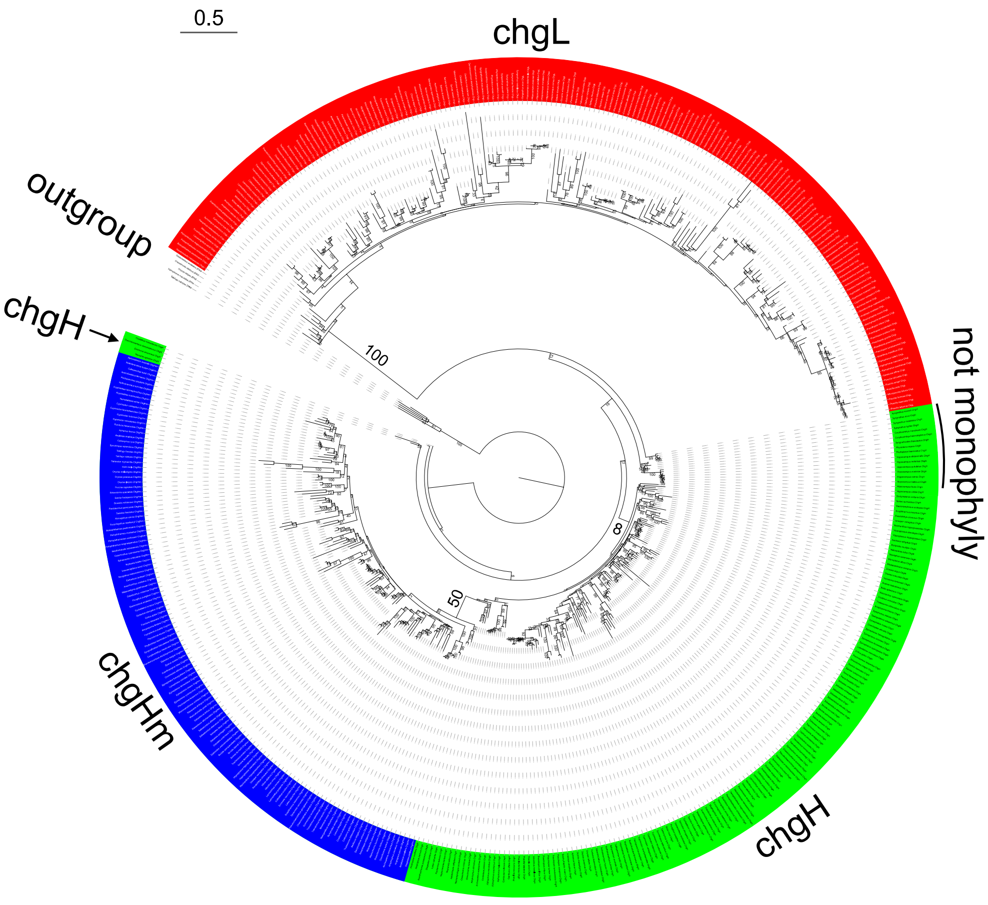

Figure S2C

MOLECULAR ECOLOGY

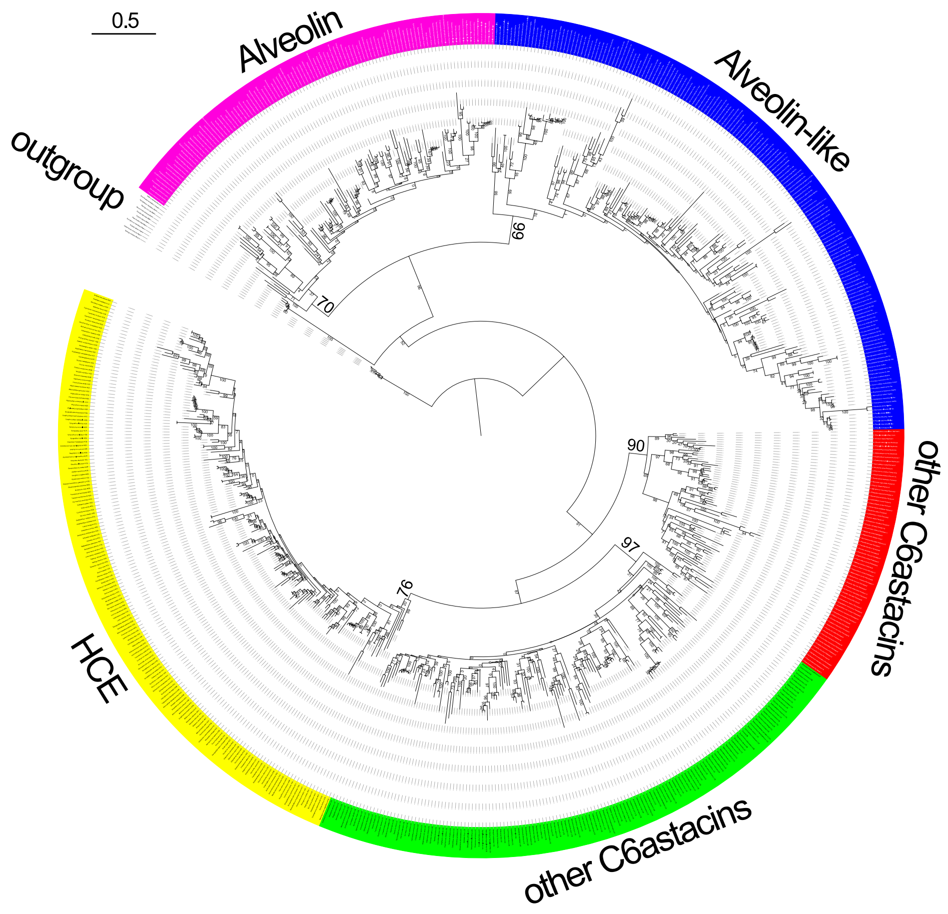

Figure S2D

Fig. S2 Molecular phylogenetic trees with all bootstrap values
Neighbor-joining phylogenetic trees with all bootstrap values added to the same molecular phylogenetic trees as (A) choriongenins and (B) astacins, as shown in Fig. 2. Maximum-likelihood phylogenetic tree of (C) choriogenins and (D) astacins.

MOLECULAR ECOLOGY

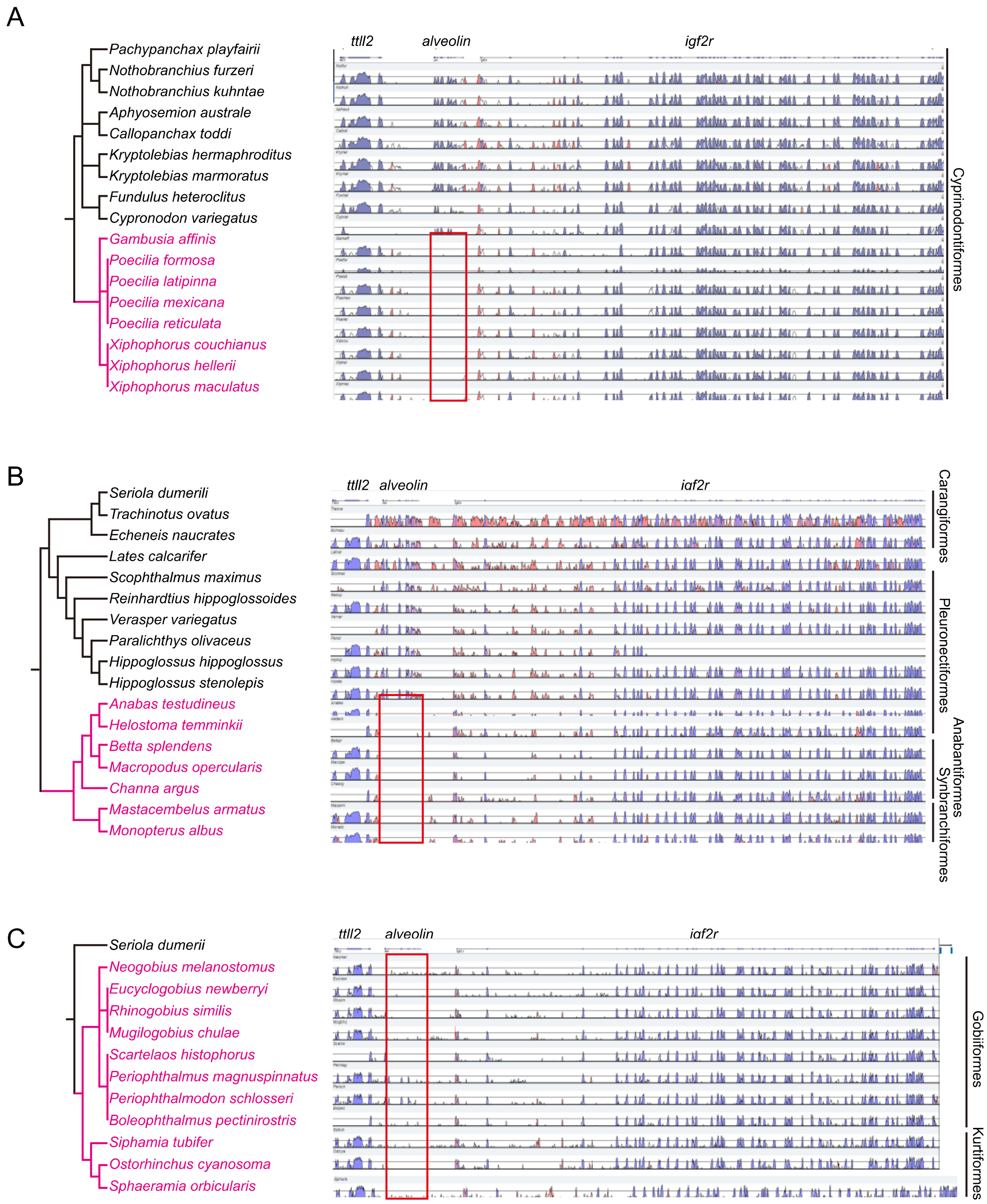

Figure S3

MOLECULAR ECOLOGY

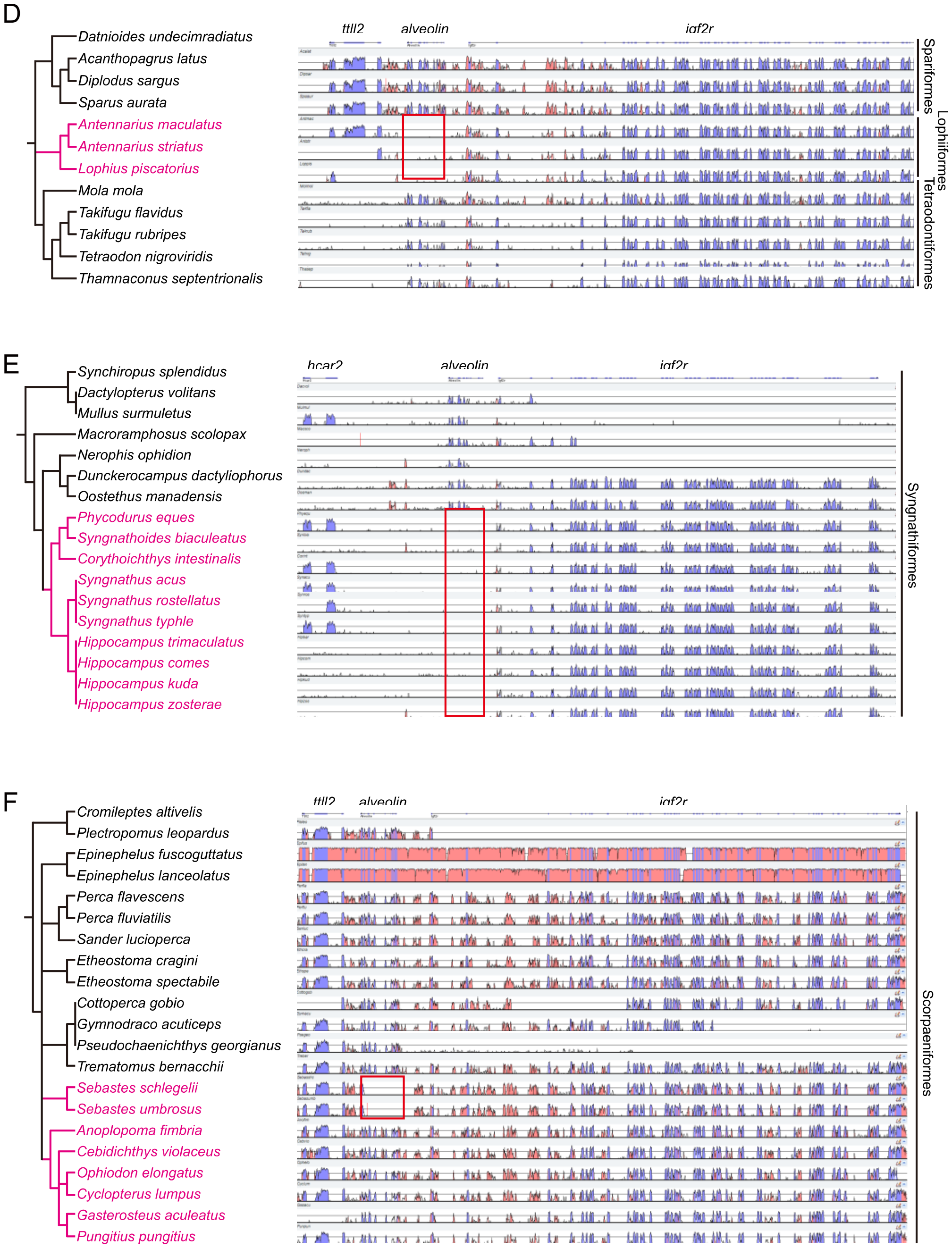

Figure S3

MOLECULAR ECOLOGY

Fig. S3 Visualization of the gene loss pattern of the alveolin genes in egg-guarding species using a VISTA plot

The multiple global alignments of alveolin genes in (A) Cyprinodontiformes, live-bearers; (B) Anabantiformes and Synbranchiformes, bubble-nest; (C) Gobiiformes, mud-nest, and Kurtiformes, mouth-brooder; (D) Lophiiformes, covered by gelatinous egg veil; (E) Syngnathiformes, nursing in a brooding pouch; and (F) Scorpaeniformes, live-bearers, were visualized using a VISTA plot. The phylogenetic relationships of the species are shown on the left (egg-guarding species colored magenta), and the top-most non-guarding species were used as a reference sequence. Conserved regions against the reference species form peaks, where blue represents exonic regions and red represents non-coding regions. The exon structure of alveolin and its flanking genes in the reference species were depicted at the top. In a region enclosed by a red box, the sequence of alveolin is nearly completely lost.

MOLECULAR ECOLOGY

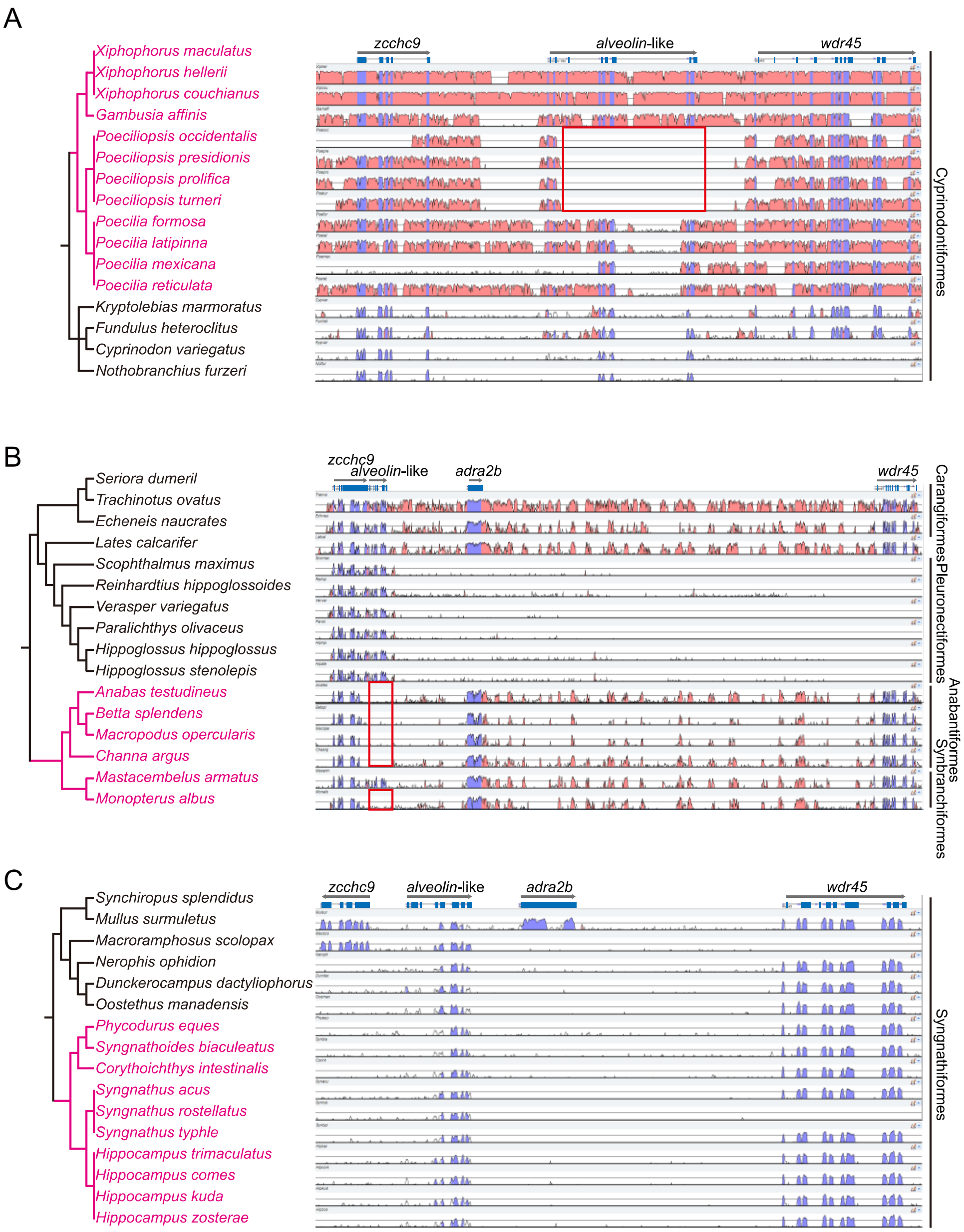

Figure S4

MOLECULAR ECOLOGY

Fig. S4 Visualization of the gene loss pattern of alveolin-like genes in egg-guarding species using a VISTA plot

The multiple global alignments of alveolin-like genes in (A) Cyprinodontiformes, (B) Anabantiformes and Synbranchiformes, and (C) Syngnathiformes were visualized as in Fig. S3 using a VISTA plot. In Syngnathiformes, the full-length sequences of alveolin-like genes were conserved in all analyzed species.

MOLECULAR ECOLOGY

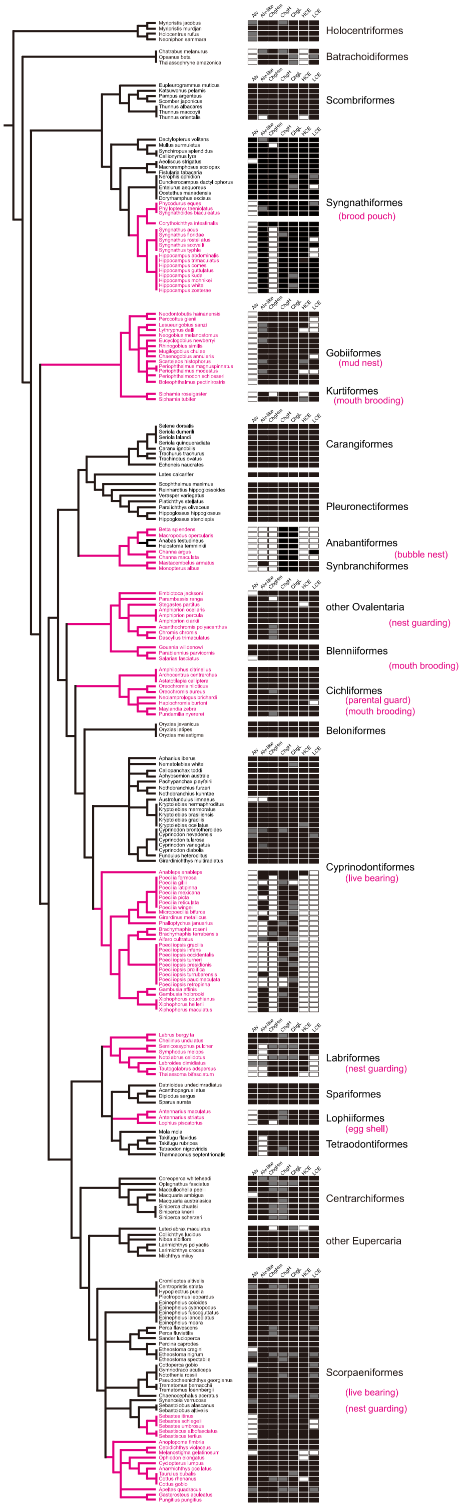

Figure S5

MOLECULAR ECOLOGY

Fig. S5 Schematic diagram of the gene loss patterns of hard-chorion–related genes in all analyzed species in this study

The presence or absence of hard-chorion–related genes identified in this study was depicted as a schematic diagram. The illustration method used is similar to that of Fig. 4, with the species names (egg-guarding species highlighted in magenta) and phylogenetic relationships shown on the left. A black square represents the presence of a full-length gene; a gray square indicates genes not detected because of genome quality; and a white square denotes clear gene loss.

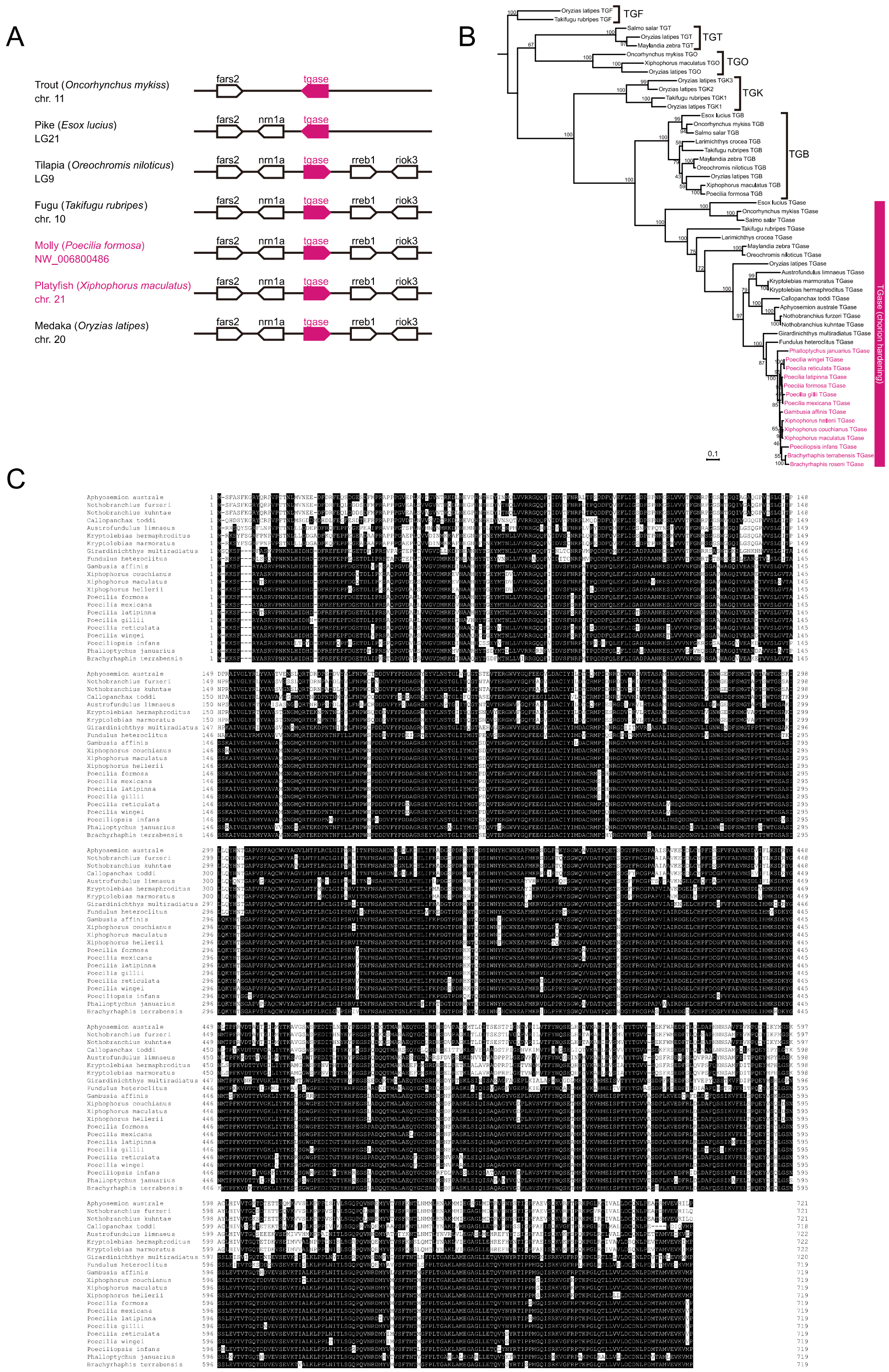
A
Trout (Oncorhynchus mykiss)
chr. 11
Pike (Esox lucius)
LG21
Tilapia (Oreochromis niloticus)
LG9
Fugu (Takifugu rubripes)
chr. 10
Molly (Poecilia formosa)
NW_006800486
Platyfish (Xiphophorus maculatus)
chr. 21
Medaka (Oryzias latipes)
chr. 20
fars2
nrn1a
tgase
rreb1
riok3
B
TGF
TGT
TGO
TGK
TGB
TGase (chorion hardening)
0,1
C

Figure S6

MOLECULAR ECOLOGY

Fig. S6 Conservation of transglutaminase genes in Cyprinodontiformes
(A) Genome synteny of transglutaminase (tgase) genes in Euteleostei. The genomic synteny of tgase genes, which have an essential role as catalysts for chorion-hardening, was illustrated using the same method as in Fig. 2 and Fig. S1. As demonstrated in our previous study (Yasumasu et al., 2024), the genomic synteny of tgase genes is well-conserved, particularly in egg-guarding Cyprinodontiformes (Molly and Platyfish; highlighted in magenta), which is one of the most significantly associated groups with hard-chorion–related genes and egg-guarding. (B) Molecular phylogenetic tree of tgase genes constructed using the most likelihood method with full-length amino acid sequences. The full-length sequences of tgase genes were well-conserved in all of the analyzed egg-guarding Cyprinodontiformes (highlighted in magenta), as depicted in the molecular phylogenetic tree constructed using the most likelihood method. (C) Multiple alignments of tgase genes in Cyprinodontiformes. Conserved sequences among species are boxed.

MOLECULAR ECOLOGY

Table S1. List of all species, gene sequences, and accession numbers used in this study.

Table S2. Binary data of gene lost and egg-care for PGLS analysis.

Table S3. List of accession number of RNA-seq data and genome assembly.

Phylogenetic tree of Cyprinodontiformes for PGLS analysis

((((((((((Poefor:0.00262783,Poelat:0.00437337)0.8630:0.00641124,Poemex:0.01005992)0.853
0:0.00610812,(Micbif:0.00000000,(Nemwhi:0.00000000,Poepic:0.00000000)1.0000:0.000000
00)1.0000:0.04573176)0.5420:0.00213597,Poereti:0.01566600)1.0000:0.02526683,(Gamaff:0
.01894408,(Xipmac:0.00380451,(Xipcou:0.00454340,Xiphel:0.00484634)0.4290:0.00083590)
0.9480:0.01129316)0.8020:0.00499819)0.9790:0.01073953,(Poeturr:0.02831660,(Poegra:0.01
268217,Poepro:0.02587682)0.4540:0.00189215)0.9520:0.01492058)1.0000:0.07797979,Anaa
na:0.08490268)0.5250:0.00664888,Girmul:0.10971151)0.3270:0.01017315,Aphibe:0.138571
28)0.4330:0.01004755,(Funhet:0.11978669,(Cypdia:0.01624914,Cyptul:0.02074644)1.0000:0
.12932296)0.4410:0.00554015,((((Notfur:0.00396767,Notkuh:0.00357954)1.0000:0.12661360
,Caltod:0.17003536)0.9960:0.05531923,Pacpla:0.13962126)0.8690:0.01982658,(Auslim:0.08
427622,((Krybra:0.01644956,Krygra:0.00753241)0.9810:0.02172668,(Kryoce:0.00933735,(K
ryher:0.00173543,Krymar:0.00294842)0.9070:0.00481391)1.0000:0.04406688)0.9940:0.0319
9829)1.0000:0.07835590)1.0000:0.11191318);

Phylogenetic tree of Anabantiformes for PGLS analysis

((((((((Craign:0.01653759,Seldor:0.02844041)0.5000:0.00167259,Tratra:0.02934573)1.0000:0
.04011428,Traova:0.11313805)0.4860:0.00830491,(Serdum:0.00536049,(Serlal:0.00618909,S
erqui:0.00547966)0.9720:0.00636042)1.0000:0.03905297)0.7000:0.00941604,Echnau:0.0942
4754)0.5870:0.01189110,Latcal:0.06695987)0.5060:0.00297716,(Scomax:0.11766992,(Paroli
:0.04551375,(Plaste:0.03635503,(Reihip:0.00061445,(Vervar:0.00000000,(Hiphip:0.0022769
5,Hipste:0.00000000)1.0000:0.01897081)0.5650:0.00645914)0.4750:0.00510328)0.8560:0.01
125384)0.9990:0.05404686)0.7760:0.02186618)0.8630:0.02298751,(Masarm:0.08958896,(Ch
aarg:0.00955517,Chamac:0.01796950)1.0000:0.09210285)0.5400:0.01635426,((Anates:0.070
90978,Helbur:0.07869544)0.9440:0.01987427,(Monalb:0.12557365,(Betspl:0.11119289,Mac
ope:0.06280956)0.9990:0.05387363)0.3920:0.00728665)0.2680:0.00612999);

Phylogenetic tree of Syngnathiformes for PGLS analysis

((((((((((Hipcom:0.01013589,Hipmoh:0.03130130)0.4520:0.00327457,(Hipkud:0.00000000,H
ipwhi:0.00000000)1.0000:0.02136468)0.3020:0.00264753,Hipgut:0.00944776)0.3090:0.0030
2468,Hipabd:0.04505000)0.4500:0.00519301,Hipzos:0.01809419)1.0000:0.08117491,(Synbia
:0.06164760,(Phyequ:0.00796257,Phytae:0.00632338)1.0000:0.02793905)0.9980:0.05092593
)0.3550:0.00595994,(Synacu:0.01503985,(Synsco:0.04083261,(Synros:0.00110447,Syntyp:0.
00267626)0.9760:0.02210686)0.6870:0.00566682)1.0000:0.08950630)0.4050:0.01059658,Co
rint:0.12008707)0.9920:0.04377022,((Entaeq:0.02223770,Neroph:0.01386730)1.0000:0.0974

MOLECULAR ECOLOGY

0023,(Oosman:0.04835157,(Dundac:0.02626513,Dorexc:0.04400406)0.5210:0.00379270)0.8
330:0.01352690)0.9970:0.03932796)0.8820:0.02687196,(Aeostr:0.10304415,(Fistab:0.08961
590,Macsco:0.06689327)0.6560:0.01067291)0.6220:0.01396413,Synspl:0.30711829);

Phylogenetic tree of Lophiiformes for PGLS analysis

(((((Dipsar:0.01654252,Spaaur:0.00000000)0.9270:0.00532514,Acalat:0.01067930)0.9970:0.0
4049130,Datund:0.03350317)0.5210:0.00697948,(Antmac:0.00907452,Antstr:0.01133435)1.0
000:0.09405278)0.5250:0.01391682,Loppis:0.10759298,(Thasep:0.14130492,(Tetnig:0.08675
493,(Takfla:0.00000000,Takrub:0.00595659)1.0000:0.07086068)0.9900:0.05555817)0.8530:0
.03052932);

>Anaana
MEFSFDISQLFSERISILDNNLVASPQTNERPDLQAKIATVIDELGRASAKAQKLPAPITSASKLQSQKHQLYLMKDGESSRGRGVVVGFL
KVGYKKLFLLDRNGVHVEAEPLCVLDFYVAENLQRHGYGLELFNFMLQHQNMEPVLMAYDRPSTKLLAFLAKHYSLKQSVPQVNNF
VAFEGFFHKRTVAQLRRVKKPDGEIKPYSLMEREVVRQDQRTLPWPFAAPQSPQRSVSSQCSQSPSAGSSPSRVLPSVTHSSFPGDNREQ
SPHSPLMDCCRTRRSNSQKGLVARSSLYSRHMDIRIPGQLDRNVSGLRPTEDQTGALGWKDEHRSQPGSSPPLVPQSNDVCSLTTIKGIK
GDLDSSATDTKLLDNKQSSPKGNYKSLEKENARKPQDWSWTVGGTYSTAQWVKRNQEYRSTRPW
>Aphibe
MEFPFDINQIFPERISILDENLVASCHSKERPDLHAKISTVIDELGKASAKAQQLPAPITSASKLQSQKHQLYLMKNGEGSRGRGLVVGFL
KVGYKKLFLLDRNGAHVEVEPLCVLDFYIAENLQRHGYGQELFNFMLQHQIVEPVLMAYDRPSTKLLAFLAKHYSLRQSVPQVNNFV
AFEGFFHKRTVAQSRRVRKPGGEIKPYSLMEREVVHQEQRTLPWPFAAPQSPQPSISSQCSQSPSVGSSPNRVLPPVTHSSFPGDNRQQTR
QSPLMECCRTSRSNGQKAIVARSSLYSWCMDIRVAGFLDRNLSGKHFLCPTRFQSGLSSLLVPQSNDICRLTTIDGIKGHLDSAAKSHAT
AEEIRVFDSRQSSSEGNHKPLEKENARKQQDWSWTVGGNTSTAQWVKQSQKCRNTCPW
>Auslim
MDFPFDINQLFSERISVLDQNLVASWRSTGSPDLQSHIATVIDELGIASAKAQQLTAPITSASKLQSQDHQVYLLKDRQSCGARGVAVGF
LKVGFKKLFLLNQQGVHIEAEPLCVLDFYVAENLQRHGYGLELFNFMLQHKNVEPVEMAYDRPSPKLLAFLAKHYSLMQSVPQVNNF
VVFEGFFLNRTAVQLRKAPYKKPDGEIKPYSLMEREVVHQEQRTLPWPFAQPQSPQRLVSSQRSHSVSVGSSPSRVLPPSSSPGSSREQS
VQLPHIERCRTRRSNQQGLAARSSLYSRHMDSRTLGLLDLSGVRPVGRETFRWAGVHTPQSRFQPLGLPSLLVSKNGANNHTTIDGKGD
PGSAAGSCDATEEITLMVHQQSSAHDNQQPLKKEGARNHQDWSWVVGSNCSTALCVKQKQGYRSTRPW
>Caltod
MDFPFDINQLFSDRISILDRNLVASHRSTERPDLQNQIAAVIDELGRASAKAQHLAAPVTNASKLQTQDHQLFLLKDRESSGGRGAAVGF
LKVGYKKLFLLDQHGAHIEAEPLCVLDFYVAETLQRHGYGLELFSFMLQNKNLEPVLMAYDRPSDKFLAFLAKHYCLTQSIPQVNNFV
VFEGFFLDKSVQLRRTPLKKPDGDIKPYSLVERDVLHQEQRTLPWPFAPPQSPQRSVPSQCSHSVRVGSSPSRIPPVAPSSPLGVSRNQAG
QLALTERCRLRSSQRGLAARSSLYSRHIDGRTAGLLGGHLSGKVPFDHLSGHVLHSNPFFFPLFYRPYPLWLPPLTASDRIRVDGHSSTGS
TLRNLDSAAELGGDAEVQLANGHKGSVDDSRGPLLLVGSWTLGANCSSAQWVKQKQGSRSTRPW
>Cypdia
MEFPFDINHIFSDRISILDHTLVASRQSAVRTDLQDQIAAVVDELGRASAKAQQLPAPITSASKLQSQKHQLYLMKDGESNRGRGLVVGF
LKVGYKKLFLLDRNGVHVEAEPLCVLDFYVAETLQRHGYGLELFNYMLQHQNVEPELLAYDRPSNKLLAFLAKHYSLVRSVPQVNNF
VAFEGFFHRRSVAQLRRVKKPDGEIKPYSLMEREVVRQEQRTLPWPFAAPQSPQRSASSQCSSSVGSSPSRAPPGVTHSSFRGDDREQSP
HSPLIDCCRTRRSNPKALVARSSLYSRHMDLRAAGLLDRNLSGKVLLGVFLFDPGFCIVFLLTRSQPAPRLPQSDDICILTSTEGHLESQEP
AEKSCMLVSKQNLCRGDQKPLKETENGRKAPDWSWTVGGSCSAARWVKQKQEYRSTRPW
>Cyptul
MEFPFDINHIFSDRISILDHTLVASRQSAVRTDLQDQIAAVVDELGRASAKAQQLPAPITSASKLQSQKHQLYLMKDGESNRGRGLVVGF
LKVGYKKLFLLDRNGVHVEAEPLCVLDFYVAETLQRHGYGLELFNYMLQHQNVEPELLAYDRPSNKLLAFLAKHYGLVRSVPQVNNF
VAFEGFFHRRSVAQLRRVKKPDGEIKPYSLMEREVVRQEQRTLPWPFAAPQSPQRSASSQCSSSVGSSPSRAPPGVTHSSFRGDNREQSP
HSPLIDSCRTRHSNPKALVARSSLYSRHMDLRAAGLLDRNLSGPGLMGDPLCSLGSQSERRSQPAPRLPQSDDICILTSTEGHLESQEPAE
KSCMLVSKQNVCRGDQKPLKETENGRKAPDWSWTVGGSCSAARWVKQKQEYRSTRPW
>Funhet
MEFPFDINQLFSERISVLDHNLVASRQSKERPDLVANIVTVVDELGRASARAQQLPAPITSASKLQSQKHQVYLMKDRESSRGSGIVVGF
LKVGYKKLFLLDRNGAHVEAEPLCVLDFYVAETLQRHGYGLELFKFMLQHQNVEPELMAYDRPSTKLLAFLAKHYSLIQSVPQVNNF
VAFEGFFHKRTVAQLRRVKKPDGEIKPYSSMEREVVRQEQRSLPWPFAPPQSPQRSVSSLSPNAGSSPSRAPPCVTHSSFPRDNREHSPQS
PLIDCCRTRRSNNQKGLVCKSSLYSRHMDIRAAGLLDRNLSGLSPAGNEARALGCKDEHRSQLGPVPLPGPLSNDICSLTLINGHLDSAA
ESKTTAEKTKMLDSKQSSSLSNQKPLLEKEKARNWQDWSWTVGGNYSTAQRVKEKQEYRSTRPW
>Gamaff
MEFPFDINQLFSERISILDQNLVASRQSKEKPDLRVKIATVLDELGKASAKAQDLPAPITSASKLQFQKHQLYLMKDGESSRGRGVVVGF
LKVGYKKLFLLDRNGVHIEVEPLCVLDFYIAENLQRHGYGLELFNFMLQHQNVEPVLLAYDRPSTKLLAFLAKHYNLRQSVPQVNNFV
AFEDFFYKRAVSQLRRVKKPDGEIKPYSLMEREAVRQEQRSLPWPFAAPQSPHHSVSSQCSQSPSAGSSPRRVLPCVTHPAFAGDSREQS

MOLECULAR ECOLOGY

PHSPLMDCCRTRRSNSEKGLVARSSLYSRHMGIRHAGLLDRNSGRTPMGDQTCALGWKNEHRAQPGLPAPPGPRSDDVYSLTTTDGTK
KHLDSAAESHATSDETKVLDNKQSSSKGNHKLLEKDNTRKQPDWSWAVGGNYSTAQWVKQNQVYRSTRPW
>Girmul
MEFHFDINQLFSERISILDQNLVASRQSKERPDLQAKIATVVDEVGRASAMAQQLPAPITSASKLQSQKHQLYLMKDVESSRGHGVVVG
FLKVGYKKLFLLDRNGVHIEAEPLCVLDFYVSELLQRHGYGLELFNFMLQHQNVEPVLMAYDRPSNKLLAFLAKHYSLMQSVPQVNN
FVAFEGFFHKRAVAHLRRVKKPDGDIKPYSLMEREVVHQEQRTLPWPFAPPHSPQRSASSQCSPSPSVGNSPSRVLPCVTHSSLPGDNSE
QSPRSPLINCCRTRRSNSQKGLVARSNLYSRHMEIRAGGLLDRNLSGLRPMGDQTCVLGCKDEHSSQPGLPPQLEPLSGHIYSLTTIDGIK
GHLDSAAESQATEEETKVLDDKRSSSESNQKPLLEKKNARKQPDWSWTVGENYSAGQWVKQKQKYRSTRPW
>Krybra
MEFPFDIKQLFSERISVLDQNLVASWRSTGSPDLQSHIATVIDELGRASAKAQQLSAPITSASKLQSQDHQLYLLKDRESCGGRGVVVGF
LKVGYKKLFLLNQQGVHIEAEPLCVLDFYVAENLQRHGYGLELFNFMLQHKNAEPVQMAYDRPSPKLLAFLAKHYSLMQSVPQVNNF
VVFEGFFLNRTAVQLRKAPHKKPDGEIKPYSLMEREVVHQEQRTLPWPFAHPQSPQRLVSSQRSHSVSVGSSPSRVLPPSSSPGSSRDQG
VQLPLIERCRTRRSNLQGLAARSSLYSRHMDSRTMGLLDRHLPDVKPVGDPTYRLVSVHTPQSRFQTLRLPSLLASEPNGACNQTSIDSK
KWDLGSAAEPCDAAEDTKVMGHQQSSPHGNRQPLLEKEGTRNQRGWLWTVGANCSTALYVKQKQGYRSTRPW
>Krygra
MEFPFDIKQLFSERISVLDQNLVASWRSTGSPDLQSHIATVIDELGRASAKAQQLSAPITSASKLQSQDHQLYLLKDRESCGGRGVVVGF
LKVGYKKLFLLNQQGVHIEAEPLCVLDFYVAENLQRHGYGLELFNFMLQHKNAEPVQMAYDRPSPKLLAFLAKHYSLMQSVPQVNNF
VVFEGFFLNRTAVQLRKAPHKKPDGEIKPYSLMEREVVHQEQRTLPWPFAHPQSPQRLVSSQRSHSVSVGSSPSRVLPPSSSPGSSRDQG
VQLPLIERCRTRRSNLQGLAARSSLYSRHMDSRTMGLLDRHLPGKLTNSCTLSLPTRFQTLRLPSLLASEPNGACNQTSIDSKKWDLGSA
AEPCDAAEDTKVMGHQQSSPHGNRQPLLEKEGTRNQRGWLWTVGANCSTALYVKQKQGYRSTRPW
>Kryoce
MELPFDINQSFSERISVLDQNLVASWRSTGSPELQSHIATVIDELGRASAKAQQLSAPITSASKLQSQDHQLYLLKDRESCGGRGVVVGF
LKVGYKKLFLLNQQGVHIEAEPLCVLDFYVAENLQRHGYGLELFNFMLQHKNAEPVQMAYDRPSPKLLAFLAKHYSLMQSVPQVNNF
VVFEGFFLNRTAVQLRKAPYKKPDGEIKPYSLMEREVVHQEQRTLPWPFAHPQSPQRLVSSQRSHSVSVESSPSRVLPPSASPGSSRDQG
VQLPLIERCRTRRSNLQGLAARSSLYSRHMDSRTIGLLDRHLPGVRPVGDPTYRLASVCTPHSRSQPLRLPHLLTSETDGGCSHTSVDSK
PWDLGSAAAPCDAAEDTKVMGHQQSSPHGNRQPLLETEGARTQRGWLWTAGANCSTAMCVKQKQGCRSTRPW
>Kryher
MEFPFDINQSFSERISVLDQNLVASWRSTGSPELQSHIATVIDELGRASAKAQQLSAPITSASKLQSQDHQLYLLKDRESCGGRGVVVGF
LKVGYKKLFLLNQQGVHIEAEPLCVLDFYVAENLQRHGYGLELFNFMLQHKNAEPVQMAYDRPSPKLLAFLAKHYSLMQSVPQVNNF
VVFEGFFLNRTAVQLRKAPYKKPDGEIKPYSLMEREVVHQEQRTLPWPFAHPQSPQRLVSSQRSHSVSVESSPSRVLPPSASPGSSRDQG
VQLPLIERCRTRRSNLQGLAARSSLYSRHMDSRTIGLLDRHLPGVRPVGDPTYRLASVCTPHSRSQPLRLPPLLASETDGGCSHTSVDSK
TWDLGSAAAPCDAAENTKVMGHQQSSPHGNRQPLLETEGARTQRGWWWTAGANCSTMCVKQKQGCRSTRPW
>Krymar
MEFPFDINQSFSERISVLDQNLVASWRSTGSPELQSHIATVIDELGRASAKAQQLSAPITSASKLQSQDHQLYLLKDRESCGGRGVVVGF
LKVGYKKLFLLNQQGVHIEAEPLCVLDFYVAENLQRHGYGLELFNFMLQHKNAEPVQMAYDRPSPKLLAFLAKHYSLMQSVPQVNNF
VVFEGFFLNRTAVQLRKAPYKKPDGEIKPYSLMEREVVHQEQRTLPWPFAHPQSPQRLVSSQHSHSVSVESSPSRVLPPSASPGSSRDQG
VQLPLIERCRTRRSNLQGLAARSSLYSRHMDSRTIGLLDRHLPGVRPVGDPTYRLASVCTPHSRSQPLRLPPLLASETDGGCSHTSVDSK
TWDLGSAAAPCDAAENTKVMGHQQSSLHGNRQPLLETEGARTQRGWWWTAGANCSTMCVKQKQGCRSTRPW
>Micbif
MEFPFDINQLFSERISILDQNLIASRQSKEKPDLRVKIATVLDELGKASAKAQDLPAPITSASKLQFQKHQLYLMKDGESSRGRGVVVGFL
KVGYKKLFLLDRNGVHIEVEPLCVLDFYIAENLQRHGYGLELFNFMLQHQNVEPVLLAYDRPSTKLLAFLARHYNLRQSVPQGETCHV
NNFVAFEDFFHKIAVSELRRVKKSDGDIKPYSLMEREAVRQEQKTLPWPFAAPLSPHHSVSPQSSQSPSAGSSPRRLLSCVTHPVFAGDS
REQSPHSPLMDCCRTRRSNSEKGLVARSSLYSRHMDIKHAGLLDRNSGRIPMGDQTCALGWKNGHSRARPGLAAPPGLQSDDVYSLTT
TDDTKKYLDSATEAHVTSETKVLDNKQRLPEGNRKPLEKDNTFLPMTSQLDWSWAVGGNYSTAQWVKQNQVYRSTRPW
>Nemwhi
MEFPFDINQLFSERISILDQNLIASRQSKEKPDLRVKIATVLDELGKASAKAQDLPAPITSASKLQFQKHQLYLMKDGESSRGRGVVVGFL
KVGYKKLFLLDRNGVHIEVEPLCVLDFYIAENLQRHGYGLELFNFMLQHQNVEPVLLAYDRPSTKLLAFLARHYNLRQSVPQGETCHV
NNFVAFEDFFHKIAVSELRRVKKSDGDIKPYSLMEREAVRQEQKTLPWPFAAPLSPHHSVSPQSSQSPSAGSSPRRLLSCVTHPVFAGDS
REQSPHSPLMDCCRTRRSNSEKGLVARSSLYSRHMDIKHAGLLDRNSGRIPMGDQTCALGWKNGHSRARPGLAAPPGLQSDDVYSLTT
TDDTKKYLDSATEAHVTSETKVLDNKQRLPEGNRKPLEKDNTFLPMTSQLDWSWAVGGNYSTAQWVKQNQVYRSTRPW
>Notfur
MDFPFDINQLFSERISILDQNLSASRRSMERPDLQVQISAVIDELGRASAKAQQLAAPVTSASKLQSQNHQLYLLKDRESGGGRGSAVGF
LKVGYKKLFLLDQHGVHVEAEPLCVLDFYIAENLQRHGYGLELFSFMLQHKNVEPVLMAYDRPSKKFLAFLAKHYCLVQSVPQVNNF
VVFEGFFLNRSAVHLRRTPLKKPDGEIKPYSMVAREVVHREQRTLPWPFAPPQSPQRSVPSQCSHPVRVGFSPSRASSPVAPSSPLGGSR
DQIERYREKRSSQQGLAARSKLYSRHMDGTTTGLLDRQASGVRPAVHPKGSLEFLNTHRKSVYSQTSLGSTHRYFDFAPGFCRDPEEIQ
VMKSSLDGNQEPLQEVGLWNIEANFTAAQWMKRKLASRSTRPW
>Notkuh
MDFPFDINQLFSERISILDQNLSASRRSMERPDLQVQISAVIDELGRASAKAQQLAAPVTSASKLQSQNHQLYLLKDRESGGGRGSAVGF
LKVGYKKLFLLDQHGVHVEAEPLCVLDFYIAENLQRHGYGLELFSFMLQHKNVEPVLMAYDRPSKKFLAFLAKHYCLVQSVPQVNNF
VVFEGFFLNRSAVHLRRTPLKKPDGEIKPYSMVAREVVHREQRTLPWPFAPPQSPQRSVPSQCSHPVRVGFSPSRASSPVAPSSPLGGSR
DQIERYRERRSSQQGLAARSKLYSRHMDGTTTGLLDRQASGVRPAVHPKGSLEFFNTHRKSVYSQTSLGSTHRYFDFAPGFCRDPEEIQ
VMKSSLDGDQEPLQEVGLWNIEANFTAAQWMKRKLASRSTRPW
>Pacpla
MEFPFDINQLFSERISVLDQNLVASRRSTERPDLQSHIATVIDELGRASAKAQQLTAPITSASKLQSQNHQLYLLKDRESCGGRGVVVGFL
KVGYKKLFLLDPHGVHIEAEPLCVLDFYVAENLQRHGYGLELFHFMLQNKNVEPVLMAYDRPSNKFLAFLAKHYSLTQSVPQVNNFV

VFDGFFFNRSAVQLRRTPLKKPEGEIKPYSLVEREVVRQEQQTLPWPFAPPHSPQRSQCSQSLSVGSSPYRTLPPVTSSSSLGDGRDQSVQ
LPLIERCRARRSNQRGLAARSSLYSRHLDSRTVGLLDTHLSGKLCFCSPTLTFISKLLFSVCLLTPELCPWTRSQNSTSSLTSIGSGDLDPA
AESRSVAEIKLMDSQQTSASPKHQPLLEKDGGRSGNSWMWRANFSSAQWVKQKLVYRSTRPW
>Poefor
MEFPFDINQLFSERISILDQNLIASRQSKEKPDLRVKIATVLDELGKASAKAQDLPAPITSASKLQFQKHQLYLMKDGESSRGRGVVVGFL
KVGYKKLFLLDRNGVHIEVEPLCVLDFYIAENLQRHGYGIELFNFMLQHQNVEPVLLAYDRPSTKLLAFLARHYNLRQSVPQVNNFVA
FEDFFHKRAVSQLRRVKKSDGDIKPYSLMEREAVRQEQRTLPWPFAAPLSPHRSVSSQCSQSPSAGSSPRRVLPCVTHPAFAGDSREQSP
HSPLMDCCRTRRSNSEKGLVARSSLYSRHMDIRHAGLLDRRSGRTPMGDETYALGWKNGHRATPGLAAPPGPQSDDVYSLTTTDGTK
KHLDSATEAHATSEETKVLDNKQSSSEGNCKPLEKDNTFLPMTSQTDWSWAVGGNYSTAQWVKQNQVYRSTRPW
>Poelat
MEFPFDINQLFSERISILDQNLIASRQSKEKPDLRVKIATVLDELGKASAKAQDLPAPITSASKLQFQKHQLYLMKDGESSRGRGVVVGFL
KVGYKKLFLLDRNGVHIEVEPLCVLDFYIAENLQRHGYGIELFNFMLQHQNVEPVLLAYDRPSTKLLAFLARHYNLRQSVPQVNNFVA
FEDFFHKRAVSQLRRVKKSDGDIKPYSLMEREEVRQEQRTLPWPFAAPLSPHSSVSSQCSQSPSAGSSPRRVLPCVTHPAFAGDSREQSP
HSPLMDCCRTRRSNSEKGLVARSSLYSRHMDIRHAGLLDRRSGRTPMGDETYALGWKNGHRAQPGLAAPPGPQSDDVYSLTTTDGTK
KHLDSATEAHATSEETKVLDNKQSSSEGNCKPLEKDNTFLPMTSQTDWSWAVGGNYSTAQWVKQNQVYRSTRPW
>Poemex
MEFPFDINQLFSERISILDQNLIASRQSKEKPDLRVKIATVLDELGKASAKAQDLPAPITSASKLQFQKHQLYLMKDGESSRGRGVVVGFL
KVGYKKLFLLDRNGVHIEVEPLCVLDFYIAENLQRHGYGIELFNFMLQHQNVEPVLLAYDRPSTKLLAFLARHYNLRQSVPQVNNFVA
FEDFFHKRAVPQLRRVKKSDGDIKPYSLMEREAVRQEQRTLPWPFAAPLSPHRSVSSQCSQSPSAGSSPRRVLPCVTHPAFAGDSREQSP
HSPLMDCCRTRRSNSEKGLVARSSLYSRHMDIRHAGLLDRNSGWTPMADQTYALGWKNGHSRATPGLAAPPGPQSDDVYSLTTTDGT
KKHLDSASEAHATSEETKVLDNKQSSSEGNRKPLEKDNTFLPMTSQTDWSWAVGGNYSTAQWVKQNQVYRSTRPW
>Poepic
MEFPFDINQLFSERISILDQNLIASRQSKEKPDLRVKIATVLDELGKASAKAQDLPAPITSASKLQFQKHQLYLMKDGESSRGRGVVVGFL
KVGYKKLFLLDRNGVHIEVEPLCVLDFYIAENLQRHGYGLELFNFMLQHQNVEPVLLAYDRPSTKLLAFLARHYNLRQSVPQGETCHV
NNFVAFEDFFHKIAVSELRRVKKSDGDIKPYSLMEREAVRQEQKTLPWPFAAPLSPHHSVSPQSSQSPSAGSSPRRLLSCVTHPVFAGDS
REQSPHSPLMDCCRTRRSNSEKGLVARSSLYSRHMDIKHAGLLDRNSGRIPMGDQTCALGWKNGHSRARPGLAAPPGLQSDDVYSLTT
TDDTKKYLDSATEAHVTSETKVLDNKQRLPEGNRKPLEKDNTFLPMTSQLDWSWAVGGNYSTAQWVKQNQVYRSTRPW
>Poereti
MEFPFDINQLFSERISILDQNLIASRQSKEKPDLRVKIATVLDELGKASAKAQDLPAPITSASKLQFQKHQLYLMKDGESSRGRGVVVGFL
KVGYKKLFLLDRNGVHIEVEPLCVLDFYIAENLQRHGYGLELFNFMLQHQNVEPVLLAYDRPSTKLLAFLARHYNLRQSVPQVNNFVA
FEDFFHKRAVSQLRRVKKSDGDIKPYSLMEREAVRQEQRTLPWPFAAPLSPHRSVSSQCSQSPSAGSSPRRVLPCVTHPAFAGDSREQSP
HSPLMDCCRTRRSNSEKGLVARSSLYSRHMDIRHAGLLDRNSGRTSMGDETCALGWKNGHRAQPGLAALLGPQSDDVYSLTTTDGTK
KHLDSATEAHATSEETKMLDNKQSSPEGNRKPLEKDNTYLPMTSQPDWSRAVGGNYSTAQWVKQNQVYRSTRPW
>Poegra
MEFPFDINQLFSERISILDQNLVASRQSKEKPDLQVKIATVLDELGKASAKAQELPAPITSASKLQFQKHQLYLMKDGESSRGRGVVVGF
LKVGYKKLFLLDRNGVHIEVEPLCVLDFYIAENLQRHGYGLELFNFMLQHQNVEPVLLAYDRPSTKLLAFLAKHYNLRQSVPQVNNFV
AFEDFFHKRAVSQLRRVKKPDGEIKPYSLMEREAVRQEQRTLPWPFAAPQSPHRSVSSQCSQSPSVDSSPRRVLPCVTHAAFAGDSREQ
SPHSPLMDCCRTRRSNSEKGLVARSSLYSRHMDIRLAGLLDRNSGRTPTGDQTCTVGWKNKPSRAQPGLAALPGLQSDDVYSLTTTDG
TKKHLDSAAESHATSDETKMLDNKQSSFEGNSKPLEKDNSRKQSDWSWAVGGNYSTAQWVKQNQVYRSTRPW
>Poepro
MEFPFDINQLFSERISILDQNLVASRQSKEKPDLQVKIATVLDELGKASAKAQELPAPITSASKLQFQKHQLYLMKDGDSSRGRGVVVGF
LKVGYKKLFLLDRNGVHIEVEPLCVLDFYIAENLQRHGYGLELFNFMLQHQNVEPVLLAYDRPSTKLLAFLAKHYNLRQSVPQVNNFV
AFEDFFHKRAVSQLRRVKKADGEIKPYSLMEREEVLQEQRTLPWPFAAPQSPHRSVSSQSPSVGSSPRRVPGRGELPCVTHAAFAGDSR
EQSAHSPLMDCCRTRRSNSEKGLVARSCLYSRHMDIRLDGLLDRNSGRTPTGGQTCAVGWKNKHRAQYGLAALPGPQSDDVYSLTTT
DGTKKHLDSAAESHATSDDTKMLDNKQSSFEGNYKPLEKDNSRKQSDWSWTVGGNYSTAQWVKQNQVYRSTRPW
>Poeturr
MEFPFDINQLFSERISILDQNLVASRQSKEKPDLQVKIATVLDELGKASAKAQELPAPITSASKLQLQKHQLYLMKDGNSSRGRGVVVGF
LKVGYKKLFLLDRNGVHIEVEPLCVLDFYIAENLQRHGYGLELFNFMLQHQNVEPVLLAYDRPSTKLLAFLAKHYNLRQSVPQVNNFV
AFEDFFHKRAVSQLRRVKKPDGEIKPYSLMEREVVRQEQRTLPWPFAAPQSPHRSASSQCSQSPSADSSPKKVLPCVTHAAFAGDSKEQ
SPHSPLMDCCRTSRSNSEKGLVARSSLYSRHMDIRLAGLLDRNSGRTPTGDQTCAVGWKNKHSRAQPGLAALPRPQSDDVYSLTTIDG
TKKHLDSAAECHATSDKTKMLDNKQSSFEGNYKPLEKDHSRKQSDWSWAVGGNYSTAQWVKQNQVYRSTRPW
>Xipcou
MEFPFDINQLFSERISILDQNLVASRQSKEKPDLRVKIATVLDELGEASAKAQDLPAPITSASKLQFQKHQLYLMKDGESSRGRGVVVGF
LKVGYKKLFLLDRNGVHIEVEPLCVLDFYIAENLQRHGYGLELFNFMLQHQNVEPVLLAYDRPSTKLLAFLAKHYNLRQSVPQVNNFV
AFEDFFYKRAVSQLRRVKKPDGEIKPYSLMEREAVRQEQRTLPWPFAAPQSPHRSVSSQCSQSPSAGSSPRRVLPCVTHPAFAGDSREQS
PHSPLMDCCRTRRSNSEKGLVARSNLYSRHMDIRHAGLLDRNSSWTPMGDQTCALGWKNEHSIVSLLKRAQPGLGAPPGPQSDDVYSL
TTTDGTKKHLDSAAESHATSDKTKVLDNKQSSSEGNHKPLEKDHIRKQPDWSWAVGGNYSTAQWVKQNQVYRSTRPW
>Xiphel
MEFPFDINQLFSERISILDQNLVASRQSKEKPDLRVKIATVLDELGEASAKAQDLPAPITSASKLQFQKHQLYLMKDGESSRGRGVVVGF
LKVGYKKLFLLDRNGVHIEVEPLCVLDFYIAENLQRHGYGLELFNFMLQHQNVEPVLLAYDRPSTKLLAFLAKHYNLRQSVPQVNNFV
AFEDFFHKRAVSQLRRVKKPDGEIKPYSLMEREAVRQEQRTLPWPFAAPQSPHRSVSSQCSQSPSAGSSPRRVLPCVTHPAFAGDSREQS
PHSPLMDCCRTRRSNSEKGLVARSNLYSRHMDIRHAGLLDRNSSWTPMGDQTCALGWKNEHSIVSLLRRAQPGLAAPPGPQSDDVYSL
TTTDGTKKHLNSATSDKTKVLDNKQSSSEGNHKPLEKDHIRKQPDWSWAVGGNYSTAQWVKQNQVYRSTRPW
>Xipmac

MEFPFDINQLFSERISILDQNLVASRQSKEKPDLRVKIATVLDELGEASAKAQDLPAPITSASKLQFQKHQLYLMKDGESSRGRGVVVGF
LKVGYKKLFLLDRNGVHIEVEPLCVLDFYIAENLQRHGYGLELFNFMLQHQNVEPVLLAYDRPSTKLLAFLAKHYNLRQSVPQVNNFV
AFEDFFYKRAVSQLRRVKKPDGEIKPYSLMEREAVRQEQRTLPWPFAAPQSPHRSVSSQCSQSPSAGSSPRRVLPCVTHPTFAGDSREQS
PHSPLMDCCRTRRSNSEKGLVARSNLYSRHMDIRHAGLLDRNSGWTPMGDQTCALGWKNEHSIVSLLRRAQPGLAAPPGPQSDDVYS
LTTTDGTKKHLDSAAESHATSDKTKVLDNKQSSSEGNHKPLEKDHIRKQPDWSWAVGGNYSTAQWVKQNQVYRSTRPW

>Anates
MEFPFDINQIFSERISILDQTLVASRKSAGRPDLQANTATVIDELGRASAKAQQLLAPITSASKMQSQKHQLYLMKDGERNGGRGVVVG
FLKVGYKKLFLLDRQGVHIEAEPLCVLDFYIAENLQRHGYGLELFEFMLQHKNLEPVMMAYDRPSPKFLSFLAKHYCLMQSVPQVNNF
VVFEGFFLNRAAAQLRKIPLKKPDGEIKPYSLMEREVVRHEQRALPWPFAPPHSPQRSVSSQCSHSLSVGSSPSRAPARAAQASAPGSNR
DQTPQSLLTERCRARRTNQQGLVARCSLYSRHMDNRAVGLLDRHLPGLRPLGDQSHALEYTDTHRLASIHTPQCRSQALSPPPLSASKK
DGICSQTSLHSREKHQVPGAEACSAEEEIRLLDIPQSPPGSKFANQRHQLEKRSNTGQQGWSWTVGENCYTAQWVKQKQEYRSTRPW
>Betspl
MEFPFNINHLFSERISVLDEVLVAGRKSVGRPDHQANIATVIDELGKASAKAQQLPASITSASKMQSQKHQLYLLKDGERNGGRGVLVG
FLKVGYKKLFLLDQQGVHIEAEPLCVLDFYIAESLQRHGYGLELFDFMLQHKNLEPVMMAYDRPSPKFQSFLSKHYCLTQSVPQVNNF
VVFEGFFLNRAAAQLRKVPLKKTDGEIKPYSLLEREGKHLSVRQEQRALPWPFAPPHSPQLLVSSQFSHPQSTASSPSKAATRSDPASAL
GSSREQSPQSPLIERCRARRTNQRCLVAKCVLYSRHMDSRDVGPMEGHLPGVRPVGDKSHTDTHSLSSTHTLQSRSQALLPLSASKDGI
RSLTFLSSKDTHLDTKSKHSCSKGVTQPLAGNMFVNQRVMERKSQTSQQGWSWTVGENCCTAQSVKQKQEYRGTRPW
>Chaarg
MEFPFDINHLFSERVTVLDQNLVAGRKTAGRPDLQANTATVIDELGKASAKAQQLPAPITSASKLQSQKHQLYLLKDGESNGGRGVVV
GFLKVGYKKLFLLDRQGVHIEAEPLCVLDFYVAENLQRHGYGLELFDFMLQHKNLEPVTMAYDRPSPKFLSFLAKHYCLTESVPQVNN
FVVFEGFFLNRAVAQLRKVPLKKPDGEIKPYSVIEREAVRQEQRALPWPFAPPHSPQRLVSSQYSHSLSVGSSPSRAPAQAALASAPGSN
RDQNPQSPQIERCRARRTSKQGLVARCSLYSGDMDSRAAVLLDNHFPGLRTVGDQSYPTGYTDKRRVASFHTPQFRSQALSLPPLPVSK
KNGICSQTSLKSRKTHQEDSGVEARSAEEIKVLDFPHCNAQSVVNKRHLLEKESNRSQGDWSWTLGVNCYTTQWVKQKQEYRSTQPW
>Chamac
MEFPFDINHLFSERVTVLDQNLVAGRKTAGRPDLQANTATVIDELGKASAKAQQLPAPITSASKLQSQKHQLYLLKDGESNGGRGVIVG
FLKVGYKKLFLLDRQGIHIEAEPLCVLDFYVAENLQRHGYGLELFDFMLQHKNLEPVTMAYDRPSPKFLSFLAKHYCLTESVPQVNNFV
VFEGFFLNRAVAQLRKVPLKKPDGEIKPYSVIEREAVRQEQRALPWPFAPPHSPQRLVSSQYSHSLSVGSSPSRAPAQAALASAPGSNRD
QNTQSPLIERCRARRTSKQGLVARCSLYSGDMDSRAAVLLDNHFPVLRTVGDQSHATGYTDKRRVASFHTPQIRSQALSLPPLPVSKKN
GICSQTSLKSRKTHQEDSGVEARSAEEIRVLDFPHCNTQGVVNKRHLLEKESNRSQQDWSWTLGVNCYTTQWVKQKQEYRSTQPW
>Helbur
MEFSFDINHLFSERISILDQNLVAGRKSTGRPDLQANTATVIDELGRASAKAQQLPAPITSASKLQSQKHQLYLLKDGERNGGRGVVVGF
VKVGYKKLFLLDRQGVHIEAEPLCVLDFYIAENLQRHGYGLELFNFMLQHKNLEPVMMAYDRPSPKFLSFLAKHYCLTQSVPQVNNFV
VFEGFFLNRAAAQLRKVPLKKPEGEIKPYSLMEREVVRQEQRVLPWPFAPPHSPQRSVSSQCSQYRSVGSSPSRAAVPAPAPGSNRDQSP
QSPLNERCRARRTNQQGLVASCSLHSRHMDSRDVAQLERHLPGLRPLGDQSHALGCTDTHRLASFHTPQSRSQAVSLPPLSAFKKGGD
CSRTSLNSRVTSHLNPGAEACDAEEEIRVLNIPQSARSMFVNQRHVSEKQRDTSQQGWSWTVGEKCYTAQWVKQKQEYRSTRPW
>Macope
MEFPFDINQLFSERISVLDQALVAGRNSVGRPELQANIATVIDDLGRASAKAQQLPASITSASKMQSQKHQLYLLKDGERNGGRGVIVG
FLKVGYKKLFLLDQQGVHVEAEPLCVLDFYIAENLQRHGYGLELFDFMLQHKNLEPVTMAYDRPSPKFLSFLSKHYCLTQSVPQVVNN
FVVFEGFFLNRAAAQLRKVPLRKPDGDIKPYSLVEREAVRQEQRARPWPFAPPHSPQLSGSSQFSHSLSVGSSPSSSALGRNRDQSPQSPL
IERYRTRRTNQQGLFAKCVLYSRHMDSRDVGLLKGHLPGVRPVGDKSYTDTHRLASTHTPQSRSQTLSHLPLAASKDGICSLASSSRNN
HQQMSDNPQSLAGNMIVNQRALERKSQRDWSWTVEKNFCTAQWVKQKQEYRSTRPW
>Masarm
MEFSFDINYLFSERVTVLDQTLVPCRKSAGRPDLQAHVAKVIDELGIASAKAQQLTAPVTSASKMQAQRHQLYLLKDCKSNGGSGVVV
GFLKVGYKKLFLLDLQGVHIEAEPLSVLDFYIVENLQRHGYGLELFDFMLKHKNLEPVMMAYDRPSPKLLSFLAKHYCLTQSVPQVNN
FVVFEGFFLNRAVAQLRKVPSKKPDGEIKPYSLMEREVVRQEERALPWPFVPPHSPQRLISSQCSHSLSVGSSPNRVLSRAASASAPGSN
RDQNPQFPVIERCRARRTNQQGLVVRCSLQSPHGQQRGWTA
>Monalb
RPDLQAHVATVIDELGRASAKAQHLPAPVTSASKLQSQRHQLYLLKDGESNGGRGVVVGFLKVGYKKLFLLDQQGAHIEAEPLSVLDF
YVAENLQRHGYGRELFDFMLQHKNVEPVMMAYDRPSPKFLSFLAKHYCLTQSVPQVQSALFSFLYCVSSTATQSRKVPLRKPDGEIKP
YSLMEREVVRQEQKAPPWPFVPPHLPQESVSSQRSHSLSVESSPSRAATRAALASAPGSNRDQSPRFPLTERCREKRT
>Hiphip
MAFPFDINQLFPERVTLLDQTLVAENRKSTERPDLLAHIVTVIDELGRASAKAQQLTTPITSASKLQSQKHQLYLLKDVESNRGHGMVV
GFLKVGYKKLFLLDRQGVHIEAEPLCVLDFYIAENFQRHGYGSELFDFMLQHNNLEPVLMAYDRPSAKFLSFLAKNYWLTQSVPQVNN
FVVFEGFFLNKTVAQLRKVPLKKPDGEIKPYSVMEREVVNQEQRALPWPFPLPHSPQRSVSSQSSNSLSVGSSPSRVPLQVTSASAVAGN
RDNSPQSPLTEHCRARRTSQQGLAARCSLYHWHLDTRAVGLLDRHTHARHVLL
>Hipste
MAFPFDINQLFPERVTLLDQTLVAENRKSTERPDLLAHIVTVIDELGRASAKAQQLTTPITSASKLQSQKHQLYLLKDVESNRGHGMVV
GFLKVGYKKLFLLDRQGVHIEAEPLCVLDFYIAENFQRHGYGSELFDFMLQHNNLEPVLMAYDRPSAKFLSFLAKNYWLTQSVPQVNN
FVVFEGFFLNKTVAQLRKVPLKKPDGEIKPYSVMEREVVNQEQRALPWPFPLPHSPQRSVSSQSSNSLSVGSSPSRVPLQVTSASAVAGN
RDNSPQSPLTEHCRARRTSQQGLAARCSLYHWHLDTRAVGLLDRHTHAR
>Paroli
MAFPLDINQLFPERVTLLDQTLVAENRKSTERPDLLAHIVTVIDELGRASAKAQQLTTPITSASKLQSQRHQLYLLKDGDSNRGHGIVVG
FLKVGYKKLFLLDRQGVHIEAEPLCVLDFYIAENFQRHGYGSELFDFMLQHKNLEPALMAYDRPSPKFLSFLAKNYWLTHSVPQVNNF

VVFEGFFLNRTVAQLRKVPLKKPDGEIKPYSVMEREVVNQEQRALSWPFPPPHSPQQSVSSQSSNSLSGGSSPSRVPLQVSSASAVVGNR
DDSPQSPLIERCRARRTSSLNRSQLGFH
>Plaste
MAFPFDINQIFLERVTLLDQTLVAENRKSTERPDLLEHIVTVIDELGRASAKAQQLTTPITSASKLQSQKHQMYLLKDVESNRGHGMVV
GFLKVGYKKLFLLDRQGVHIEAEPMCVLDFYIAENFQRHGYGSELFDFMLQHNNLEPVLMAYDRPSPKLMSFLAKKYWLTQSVPQVN
NFVVFEGFFLNKTVAQLRKVPLKKPDGEIKPYSVMEREVVNQEQRALPWQFPLPHSPQRSVSSQSSNSLSVGSSPSRVPLQVTSASAVV
GNRDNSPQSPVTERCRARRT
>Reihip
MAFPFDINQLFPERVTLLDQTFVAENRKSTERPDLLAHIVTVIDELGRASAKAQQLTTPITSASKLQSQKHQLYLLKDVESNRGHGIVVG
FLKVGYKKLFLLDRQGVHIEAEPLCVLDFYIAENFQRHGYGSELFDFMLQHKNLEPVLMAYDRPSPKFLSFLAKNYWLTQSVPQVNNF
VVFEGFFLNKTVAQLRKVPLKKPDGEIKPYSVMEREVVNQEQRALPWPFPLPHSPQRSVSSQSSNSLSVGSSPSRVPLQVTSASAVAGNR
DNSPQSPLTERCRARRT
>Scomax
MEFPFDINQLFSERICFLDQTFAAENGQSAARSDLQAHIVTVIDELGRASAKAQQLTAPITSASKLQSQFHQLYLLKDGESNRGHGVVVG
FLKVGYKKLFLLDRRGVHIEAEPLCVLDFYIAENLQRHGCGLDLFDFMLQHKNLEPELMAYDRPSHKFLSFLAKHYCLIHSVPQVNNFV
VFEGFFLNKTAVPLRKVPLKKPDGEIKPYSLVEREVVHQEQRTLPWPFVPPHSPQRLVSSQCSDSLSAGSSPSKVPLQVTAASALAGNRD
QSPHSPVIERSSARRTSSLTRSQLGFH
>Vervar
MAFPFDINQLFPERVTLLDQTLVAENRKSTERPDLLAHIVTVIDELGRASAKAQQLTTPITSASKLQSQKHQLYLLKDVESNRGHGMVV
GFLKVGYKKLFLLDRQGVHIEAEPLCVLDFYIAENFQRHGYGSELFDFMLQHKNLEPVLMAYDRPSPKFLSFLAKNYWLTQSVPQVNN
FVVFEGFFLNKTVAQLRKVPLKKPDGEIKPYSVMEREVVNQEQRALPWPFPLPHSPQRSVASQSSNSLSVGSSPSRVPLQVTSASAVAG
NRDNSPQSPLTERCRARRT
>Craign
MEFPFDINQLFSERVSILDHTLVAGRKSAGRPDLQAHIATVIDELGRASAKAQQLTAPITSASKLQSQRHQLYLLKDGERNGGHGAVVG
FLKVGYKKLFLLDRQGVHVEAEPLCVLDFYIAENLQRHGYGLELFDFVLQHKNLEPVLMAYDRPSPKFLSFLAKHYCLTQSVPQVNNF
VVFEDFFLNRAAAQLRKAPLKKPDGEIKPYSLMEREVVRQEQRARPWPFAPPHSPQRSVSSQCSHSLSVGSSPSRAPLRAAPTTTLLGDN
RDQSPQSPHMERCRARRTSQQGLVARCSLYSRHLDSKAIDLLDTRLPGLRPVGDQSHALGYTDTHRLASTHTPLSSFQPSSSKKDGVCS
QTSLNNRDTQLDTDAEEEIRTSHSPQDAAGDTLVKQRQLSEKESGRGRGGWSWTVGENCYTAQWVKQKQEYRSTRPW
>Echnau
MEFPFDINQLFSERVTILDHTLVAGRKSAGRPDLQALITTVIDEIGRASAKAQQLTAPITSASKLQSQRHQVYLLKDGERNGGHGVVVGF
LKVGCKKLFLLDRQGVHIEAEPLCVLDFYIAENVQRHGYGLELFDFMLQHKNLEPVLMAYDRPSIKFLSFLAKHYCLTQSVPQVNNFV
VFEGFFPNRTAAQLRKVPLKKQDGEIKPYSLMEREVIRQEQRALPWPFAPPHTPQRSVSSQCSHSLSVGSSPSGAPLRALRDNRDKSPQS
TLAERCRARRTSQQGLVARRCLYSRHLDSRAVELDAHLPGITPVGDQSYGLGYTDTHRLASNHTPRSRFVPLSSKKEGICSQTSLNSRDT
QPDTDAEAYSADQQIGVLDSLQGPAGRTLVNQRPLLEKESDKSQQSWSWTVGQNCYTAQWVKQKQEYRSTRPW
>Seldor
MEFPFDINQLFSEKVSILDHTLVAGRKSAGRPDLQEHIATVIDELGRASAKAQQLTAPITSASKLQSQRHQLYLLKDGERNGGHGAVVG
FLKVGCKKLFLLDRQGVHVEAEPLCVLDFYIAENLQRHGYGLELFDFVLQHKNLEPVLMAYDRPSPKFLSFLAKHYCLTQSVPQVNNF
VVFEGFFLNRAAAQLRKTPLKKPDGEIKPYSLMEREAVRQEQRARPWPFAPPHSPQRSVSSQCSHSLSVGSSPSRAPLRAAPTTLLGDSR
DQSPQSPHMERCRARRTSQQGLVARCSLYSRHLDSKVIDLLDTRLPGLRPLGDQSYALGYTDTHRLASTHTPLSSFQPPSSKKDGVCSQ
TSLNSRDTQLDTDAEEEIRTSQSPQGPAADTLVKQRQLSEKERDRGRGVWSWTVGENCYTAQWVKQKQEYRSTRPW
>Serdum
MEFPFDINQLFSERVSILDQTLVAGRKSAGRPDLQAHIATVIDELGRASAKAQQLTAPITSASKLQSQRHQLYLLKDGERNGGRGVVVG
FLKVGYKKLFLLDRQGVHVEAEPLCVLDFYIAENLQRHGYGLELFDFVLQHKNLEPVLMAYDRPSPKLLSFLAKHYCLTQSVPQVNNF
VVFEGFFLNREAAQLRKVPLKKPDGEIKPYSIMEREVVRQEQRALPWPFAPPHSPQRSVSSQCSHSLSVGSSPSRVPLRAAPTSALGANR
DQSPQSPLVERCRAKRTSQQGLVARCSLYSRHLDSRAVGLLDSHLLGMRPVVDQSYALGYTDTHRLASIHTPLSRFQPPSSKKDGLCSE
TSLNSRETQLDTDAEEISVSDSPQGPAGGMLLNHRHLLEKESGSSLRGWSWTVGEHCYTAQWVKQKQEYRSTRPW
>Serlal
MEFPFDINQLFTERVSILDQTLVAGRKSAGRPDLQAHIATVIDELGRASAKAQQLTAPITSASKLQSQRHQLYLLKDGERNGGRGVVVG
FLKVGYKKLFLLDRQGVHVEAEPLCVLDFYIAENLQRHGYGLELFDFVLQHKNLEPVLMAYDRPSPKLLSFLAKHYCLTQSVPQVNNF
VVFEGFFLNREAAQLRKVPLKKPDGEIKPYSIMEREVVRQEQRALPWPFAPPHSPQRSVSSQCSHSLSVGSSPSRVPLRAAPTSALGANR
DQSPQSPLVERCRAKRSSQQGLVARCSLYSRHLDSRAVGLLDSHLLGMRPVVDQSYALGYTDTHRLASIHTPLSRFQPPSSKKDGLCSE
TSLNSRETQLDTDAEETSASDSPQGPAGGVLLNHRHLLEKESDRSLRGWSWTVGEHCYTAQWVKQKQEYRSTRPW
>Serqui
MEFRFDINQLFSERVSILDQTLVAGRKSAGRPDLQAHIATVIDELGRASAKAQQLTAPITSASKLQSQRHQLYLLKDGERNGGRGVVVG
FLKVGYKKLFLLDRQGVHVEAEPLCVLDFYIAENLQRHGYGLELFDFVLQHKNLEPVLMAYDRPSPKLLSFLAKHYCLTQSVPQVNNF
VVFEGFFLNREAAQLRKVPLKKPDGEIKPYSIMEREVVRQEQRALPWPFAPPHSPQRSVSSQCSHSLSVGSSPSRVPLRAAPTSAHGANR
DQSPQSPLVERCRAKRTSQQGLVARCSLYSRHLDSRAVGLLDSHLLGMRPVVDQSYALGNTDTHRLASIHTPLSRFQPPSSKKDGLCSE
TSLNSRETQLDTDAEETSASDSPQGPAGGVLLNHRHLLEKESDRSLRGWSWTVGEHCYTAQWVKQKQEYRSTRPW
>Traova
MEFPFDINHLFPERVSILDQTLVAGRKSAGRPDLQAHIATVIDELGRASAKAQQLTAPITSASKLQSQRHQLYLLKDGERHGYMSHPTHT
HTHTHTHTHTLFTDRQGVHVEAEPLCVLDFYIAENLQRHGYGMELFDFMLQHKNVEPVLMAYDRPSPKFLSFLAKHYCLAQSVPQVN
NFVVFEGFFLNRAAAQLRKVPLKKPDGEIKPYSSMEREAVRQEQRALPWPFAPPHSPQRSVSSQCSHSLSVGSSPSRAPLRAAPTSILGD
NRGQSLQSPLMERCRARRTSQQGLVARSSLYSRHLDSRAVGLLDTHLPGMRPVGDQYALGNTDTHRLASIHTPLSRLQPHSSKKNGICR
QTSLNSSETQLDTDAEEKLKVSDGPQGPAGGTLVNQRHLLEKESNRRQPGWSWTVGENCYTAQWVKQKQEYRSTRPW
>Tratra

MEFPIDINQLFSEKVSILDHTLVAGRKSAGRPDLQAHIATVIDELGRASAKAQQLTAPITSASKLQSQRHQLYLLKDGERNGGHGAVVGF
LKVGYKKLFLLDRQGVHVEAEPLCVLDFYIAENLQRHGYGLELFDFVLQHKNLEPVLMAYDRPSPKFLSFLAKHYCLTQSVPQVNNFV
VFEGFFLNRAAGQLRKAPLKKPDGEIKPYSLMEREVVRQEQRARPWPFAPPHSPQRSVSSQCSHSLSVGSSPSRAPLRTAPTTLLGDSRD
QSPQSPHMERCRARRTSQQGLVARCSLYSRHLDSKAVDLLDTRLPGLRPVGDQSHALGYTDTHRLASTHTPLSSFRPSSSKKDGICSQA
SLNSRDTQLHTDAEEEIGTSDGPPGPAGDTLVKQRQLSGKESNRGRGGWSWTVGENCYTAQWVKQKQEYRSTRPW
>Latcal
MEFPFDINQLFSERVSILDHSLVAGRKSAGRPDLQAHIATVIDELGRASAKAQQLTAPITSASKLQSQRHQLYLLKDGESNGGRGMVVG
FLKVGYKKLFLLDRQGVHIEAEPLCVLDFYIAENLQRHGYGLELFDFMLQHKNVEPVLMAYDRPSPKFLSFLAKHYCLTQSVPQVNNF
VVFEGFFLNRAAAQLRKVPLKKPDGEIKPYSSMEREAVRQEQRALPWPFAPPHSPQRSASSQCSHSLSVGSSPSRVPPRVVPASAPGGNR
DQSPQSPLVERCRARRTSQQGLVARCSLYSRHLDSRAVGLLDKPLTGLRPVGNQSCALGYTDTHRLASIHTPQSRLQPLPFPPLSSKKDD
IHSQTSLNSRGIHLDTDAEACGAKEKISVLESPQCLAGGMQDNQRHLLEKDSDRSRQGWSWTVGENCYTAQWVKQKQEYRSTRPW

>Aeostr
MEFPFDINHLFTERVSVLDQSLVAGRKFAARPDLQTHVETVIDDLGRASAKAQQLTAPVTSASKLQSQGHQLYLLKDGESNGGRGVVV
GFLKVGYKKLFLLDQQGLHIEAEPLCVLDFYIAENLQRHGYGLELFDFMLKHKNLEPVLMAYDRPSPKFLSFLAKHYCLAQSVPQVNH
FVVFDGFFVSRSVAHLRKAPLKKPDGEIKPYSLVEREVVRQEQKRRPWPFAPPHSPQRSVSSQYSHSLSVGSSPTRVPPQAATVSPPGAIR
DHSLQSPLIERCRARRT
>Corint
MEFSFNVNLLFPERFSILDKSLVSGRASPNRPDLQSHVATVIDELGRASAKAQKLTTPVTTAAKLQSQNHQLYLMKDGESNGGCGTIVG
FLKVGFKKLFLLDRQGLHIEDEPLCVLDFFIVENLQRKGYGLELFNFMLQDKNLEPVLMAYDRPSPKLLSFLAKHYSLTQSVPQVNHFV
VFDGFFLNKPVAQLRTIPLKKQGGEIKPYSIMEREAVRQEQRNLPWPFAPLHNPQRLVSSHSAGSSPTRTPPQSAPTSGPGVHRNQSPNFP
LIERSRERRTSQQGQVARSGLYSRHLDNKSLELLGRPLTVLKSAGGQPDISRSTDTNLLASAPLPKSRLLTHSPPSTSKGSVVCSLTSQNK
KNLHRGRNVEVADAKTDRRAPGSATRAVLENKRHLLSKEQDKSTCGFSWTVDKSCFSPQQVKQMLFSRSTCPWS
>Dorexc
MEFPFDINHLFSERVTILDQTLGAGRVSPKRPNLQSHIVTVIDELGIASAKAQRLTTPVTSANKLQSQQHQVYLMKDGESNGGRGVIVGF
LKVGYKKLFLLDQQGVHIEAEPLCVLDFFVAHNLQRHGYGLELFNFMLQDKNLEPVLMAYDRPSPKLLSFLAKHYCLTQSVPQVNHFV
VFDGFFLNKSVGQLRKVPLRKPDGEIKPYSLMEREAVRQEQRTPPWPFAPHSPQRSGFSQYSLSVGSSPSRVPPQSATVPAPGVHGDRNP
HFPLERFRARSNRVSDVAVEDSQQVGSTERVPPPYVNGLWMLGLRQWLPQSQECHAARGRLYSRYTDTKSLPVLEKPLSGLRSGHAEA
HKVSSIPGPQSRLVPHPPPSTTFIPTSLCSNAGLPQEEAPGQVLGLEKESEAAEGAAGHTSSSGWSWTGGNGCFSAQQVKQFHLNRSTCP
W
>Dundac
MEFPFDINQLFSERVTILDQTLGAGRVSPRRPNLQSHIVTVIDELGRASAKAQKLTTPVTSANKLQSQLHQVYLMKDGESNGGRGVIVGF
LKVGYKKLFLLDQQGVHVEAEPLCVLDFFVAQNLQRHGYGLELFNFMLQDKNLEPVLMAYDRPSPKLLSFLAKHYCLTQSVPQVNHF
VVFDGFFLNKSVAQLRKAPLKKPDGEIKPYSLMEREAVRQEQRTPPWPFAHPRSSQRSVFSQYSHSLSVGSSPTRVPPQAATVPAPGVH
RDQNPHFPLDRCRARSNSQQCQAARGHLHSRYTDTISFPSLGRPLSDIIFTPFLISCLKLSRRLVTHPPPSTSMTNVTCIPPSQCNRATTLRN
MEHVVGLAKEVKAAEGAAGDISSSGWSWNAGNGCFSAQQVKQIHLNRSTRPW
>Entaeq
MEFPVDINQLFSERVTILDQTLGGGRLTPRRPNLQSQLVAVIDELGKASAKAQMLTTPVTSTIKLQSQQHQVYLMKDGESNGGRGVIVG
FLKVGRKKLFLLDKKGVHVEAEPLCVLDFFIAQNLQRHGYGLELFNFMLQDKNLEPELMAFDRPSPKLLSFLAKHYCLTQSVPQVNHF
VVFEGFFLNKAVVQLRKVSLRKPDSEIKPYSIMEREVVRQEQRTPPWPFAPPHSPQRSVFSQYSHSVGSSPTRVPPQALGVPVLGIERNQS
PHFPLIERCRARSTSQRGSIARGDLHSRYMDIKSLGKRGTPLSGLRWGGERPHILGHPEAHKVANLPIPQSRLLTRPPLSTSKNNVVCCPT
FLNNRGLPHRGIGAEVVGLKKDVKAPEGATGGLLAKKQARSSSGCSWTVGNSCWSAQQVKQMHVYRSTCPW
>Fistab
MEFPFDINQLFCERVSVLDQTLIAGRKSAGRPDLQAHIATVIDELGRASAKAQQLTAPVTSASKLQSQRHQLYLLKDGERNGGRGVAVG
FIKVGYKKLFLLDQQGVHIEAEPLCILDFYIAENLQRHGYGLELFDFMLQHKNLEPVLMAFDRPSPKFLSFLAKHYSLTRSVPQVNNFVV
FEGFFINRSAAQLRKVPLKKPDGEIKPYSLMEREAVRQEQRTLPWPFALHSPRRSVSSQFSHSLSVGSSPTRAPLRAAAVSAPGGHRELG
PRFPLTDRCRAKRT
>Hipabd
MEFPFNINHLFSERFSILDKTLVAGRMSPRRPELQSHIATVIDELGRASAKAQKLTVPVTSATKMQSQHHQLYLMKDGECHGGRGAIVG
FLKIGHKKLFLLDPQGLHVEAEPLCVLDFFITENLQRHGHGQELFHFMLQHKKMEPALMAYDRPSPKLLSFLAKHYRLTQSVPQVNNF
VVFDGFFQNKSVAQPRSIAPRKPDSEIKPYSVAEREAVRQERQSLPWPFAAPQSPQRSLSVGSSPTRTGTSAPGLRRHQSPNFPLVPPSRE
RRPSQQGQLARDGLYSRHLDPKSLGTLGRPLPGVRSAQDQPGILGATETHKLAAMPKLQSRCAASPRRPTPEDNCVCGPTSPGNKKLHL
GTEADAEAGSPAGAGSSAAAPLQSQTHSADGREREGIGSVPFHSGASGIFSAQQVKRMLSSRATCPW
>Hipcom
MEFPFNINHLFSERFSILDKTLVAGRMSPRRPELQSHIATVIDELGRASAKAQKLTTPVTSATKMQSQHHQLYLMKDGESQGGRGAIVGF
LKVGYKKLFLLDPQGLHVEAEPLCVLDFFITENLQRHGHGLELFHFMLQHKKMEPALMAYDRPSPKLLSFLAKHYRLTQSVPQVNNFV
VFDGFFHNKSVAQPRSIAPKKPDGEIKPYSIAEREAVRQERRSLPWPFAAPQSPQRSLSVGSSPTRAVASAPGLRGDQSPNFPLGPTSRER
RPSQQGQVARDGLYSRHLDPKSLGALGRPLPGVRSAQDQPGILGATETHRLAAMPKLQSRCAASPRRPAPEDNGVCGPTSLGNKKLHL
GTEADAEAGSPTGAGSRATAPLQTQTHSAEGREREGIESAPSHTGASGHFSAQQVKRMLSSRATCPW
>Hipgut
MEFPFNINHLFSERFSILDKTLVAGRMSPRRPELQSHIATVIDELGRASAKAQKLTTPVTSATKMQSQHHQLYLMKDGESQGGRGAIVGF
LKVGHKKLFLLDPQGLHVEAEPLCVLDFFITENLQRHGNGLELFHFMLQHKKMEPALMAYDRPSPKLLSFLAKHYRLTQSVPQVNNFV
VFDGFFQNKSVAQPRSIAPKKPDGEIKPYSIAEREAVRQERRSLPWPFAAPQSPQRSLSVGSSPTRAVTSAPGLRGNQSPNFPLVPTSRER

RPSQQGQVARDGLYSRHLDPKSLGALGRPLPGVRSAQDQPGILGATETHRLAALPKLQSRCAASPHRPAPEDNGVCGPTSLGNKKLHL
GTEADAEAGSPTDAGSRLTAPLQSQTHPAEGREREGIESAPSHTGGNGRFSAQQVKRMLSSRATCPW
>Hipkud
MEFPFNINHLFSERFSILDKTLVAGRMSPRRPELQSHIATVIDELGRASAKAQKLTTPITSATKMQSQHHQLYLMKDGESQGGRGAIVGF
LKVGHKKLFLLDPQGLHVEAEPLCVLDFFIAENLQRHGHGLELFRFMLQHKKMEPALMAYDRPSPKLLSFLAKHYRLTQSVPQVNNFV
VFDGFFQNKSVAQPRSIAPKKPDGEIKPYSIAEREAVRQERQSLPWPFATPQSPQRSLSVGSSPTRAVASAPRLRGDQSPNFPLVPTSRER
RPSQQGQVARDGLYSRHLDPKSLGALGRPPPGVTSAQEQPGILGATETHKLAAMPKLQSRCAASPRQPAPEDNGVCGPTSLGNKKLHL
GTEADAEAGSPAGAGSGATAPLQNQTHSAEGREQEGLGSAPSHTGANGRFSDQQVKRMLSSRATCPW
>Hipmoh
MEFPFNINHLFSERFSILDKTLVTGRMSPRRPELQSHIATVIDELGRASAKAQKLTVPVTSATKMQSQHHQLYLMKDGESQGGRGAIVGF
LKVGYKKLFLLDPQGLHVEAEPLCVLDFFITENLQRHGHGLELFHFMLQHKKMEPALMAYDRPSPKLLSFLAKHYRLTQSVPQVNNFV
VFDGFFQNKSAAQPRWIAPKKPDGEIKPYSIVEREAVRQERRSLPWPFAAPQSPQRSLSVGSSPTRAVASEPWLRGDQSPNFPPVPPSRER
RPSQQGQVARDGLYSRHLDPKSLGALGRPLPGVRSAQDQPGILGATETHRLAAMPKLQSRSAASPRRPTPEDNGVCGPISLGNKKLHLG
TEADAEAGSAAGSGATAPLQSQTHSAEGGEREGMASAPSHTGADGRFSAQQLKWMLSSRATCPW
>Hipwhi
MEFPFNINHLFSERFSILDKTLVAGRMSPRRPELQSHIATVIDELGRASAKAQKLTTPITSATKMQSQHHQLYLMKDGESQGGRGAIVGF
LKVGHKKLFLLDPQGLHVEAEPLCVLDFFIAENLQRHGHGLELFRFMLQHKKMEPALMAYDRPSPKLLSFLAKHYRLTQSVPQVNNFV
VFDGFFQNKSVAQPRSIAPKKPDGEIKPYSIAEREAVRQERQSLPWPFATPQSPQRSLSVGSSPTRAVASAPRLRGDQSPNFPLVPTSRER
RPSQQGQVARDGLYSRRLDPKSLGALGRPPPGVTSAQEQPGILGATETHKLAAMPKLQSRCAASPRQPAPEDNGVCGPTSLGNKKLHL
GTEADAEAGSPAGAGSGATAPLQNQTHSAEGREQEGLGSAPSHTGANGRFSAQQVKRMLSSRATCPW
>Hipzos
MEFPFNINHLFPERFSILDKTLVAGRMSPRRPELQSHIATVIDELGRASAKAQKLTTPVTSATKMQSQHHQLYLMKDGESQGGRGAIVGF
LKVGHKKLFLLDPQGLHVEAEPLCVLDFFIAENLQRHGNGLELFHFMLQHKKMEPALMAYDRPSPKLLSFLAKHYHLTQSVPQVNNFV
VFDGFFQNKSVAQPRSIATRKPDGEIKPYSIAEREAVRQERRSLPWPFAAPQSPQRSLSVGSSPTRTVTSAPGDQSPNFPLVSTSRERRPSQ
QGQVARDGLYSRHLDPKSLGALGRPLPGVISAQDQPGILGATETHRLGALPKLQSRCAASPQRPAPEDNGVCGPTSLSNKKLHLGTEAD
AEAGSPADAGSGLTAPLQSQTHSAEGREREGMERVPSHTGANGRFSAQQVKWLLSSRATCPW
>Macsco
MEFPIDINQLFSERVSILDQTLVASRKSAGRPDLQAQIETVIDELGRASAKAQQLTAPVTSASKLQSQRHQLYLLKDGESNGGRGVVMGF
LKVGFKKLFLLDQRGVHVEAEPLCVLDFYIAENLQRHGYGLELFDFMLQHKNLEPVLMAYDRPSPKLLSFLAKNYCLTQSVPQVNNFV
VFDGFFLNRSVAQLRKCPLKKAEGEIKPYSLMEREAVRREQRTPPWPFAPPHSPRRSVSSQYSHSLSVGSSPTRAPPRAAAASAPGGNRD
QNPQSPLVERCRARRT
>Neroph
MEFPFDINQLFSERVTILDQTLGGGRLTPRRPNHQSQLVAVIDELGKASAKAQMLTTPVTSTIKLQSQQHQLYLMKDGESNGGRGVIIGF
LKVGCKKLFLLDKKGVHVEAEPLCVLDFFIAQNLQRHGYGLELFNFMLQDKNLEPELMAFDRPSPKLLSFLAKHYCLTQSVPQVNHFV
VFEGFFLNKAVVQLRKVSLRKPDGEIKPYSIMEREVVRQEQRTLPWPFAPPHSPQRSVFSQYSHSVGSSPTRVPPQALGVPVPGIDRDQS
PHFPLIERCRARSTSQRGSIARGDLHSRYMDIKSLGKRGTPLSGLRWGGERPHILGHPEAHKVANLPIPQSRLLTRPPLSTSKNNVVCCPT
FLNNRGLPHRGTGAEVVGLKKDVKAPEGAAGGLLAKKQARSSSGCSWTVGNSCWSAQQVKQMQVYRSTCPW
>Oosman
MEFPFDINHLFSERITILDQSLGAGRVSPKRPSLQSHIVTVIDELGKASAKAQKLTAPVTSANKLQSQLHQIYLMKDGESNGGRGVIVGFL
KVGYKKLFLLDQQGVHVEAEPLCVLDFFVAQNLQRHGYGLELFNFMLKDKNLEPVLMAYDRPSPKLLSFLAKHYGLTQSVPQVNHFV
VFDGFFLNKSVAQLRKVPLKKPDGEIKPYSLMEREVVRQEQRTPPWPFGPPHSPQRSVFSQYSHSLSVGCSPTRAPPQAATMPAPGVRR
DQNPHFPLERCRARSNSKQCQAARGDLYSRYMDTNSLSMVGRPLSGKRFGVKYCHVQVLCASSDLSSGHAEAHRVASVPRPQSRLVT
RPPPSTSKNNVSSSLSNRGLPHRATRAQALGLEKDGRAAEGAAGEASSSGWSWTVGSSCFSAQQVKQMHLNKSTCPW
>Phyequ
MEFPFNINPLFSERFSILDKTFVASRVSSKRPDLQSHIATVIDELGRASAKAQKLTTPVTSAAKLQSQPHQLYLMKDGESKGGRGAIVGFL
KVGSKKLFLLDQQGLHIEAEPLCVLDFFIAENLQRHGYGLELFNFMLQHKNSEPVQLAYDRPSPKLLSFLAKHYCLTQSVPQVNNFVVF
DGFFLNKSVAQSRTISLKKPDSEIKPYSIMEREAVRQERRSLPWPFAPPRSPQRLVSSQFSLSLGSSPIKSPPQAATVSAPGVHRNQSQNFP
LIERSRERRTSQQGQVARDGLYSRYMDTKSLGILGRPLPGLRSVGDQSHTLGRTERHRLASVPILQSRLVTCPPLSATKTNVVCRPTSLS
NNKLLIGIDAEVAGVQKKLRAPNGTGNTARVLLESQRYLLERERDISRSGCSWTVGNGCFSAQQVKQMLLNRSTCPW
>Phytae
MEFPFNINPLFCERFSILDKTFVASRVSSKRPDLQSHIATVIDELGRASAKAQKLTTPVTSAAKLQSQPHQLYLMKDGESKGGRGAIVGFL
KVGYKKLFLLDQQGLHIEAEPLCVLDFFIAENLQRHGYGLELFNFMLQHKNSEPVQLAYDRPSPKLLSFLAKHYCLTQSVPQVNNFVVF
DGFFLNKSVAQSRTISLKKPDSEIKPYSIMEREAVRQERRSLPWPFAPPHSPQRLVSSQYSLSLGSSPIKSPPQAATVSAPGVHRNQSQNFP
LIERSRERRTSQQGQVARDGLYSRYTDTKSSGILGRPLPGLRSVGDQSHTLGRTERHRLASVPILQSRLVTCPPLSATKNNVVCRPTSLSN
NKLQIGIDAEVAGVQKKLRASNGTGSTARVLLESQRYLLERERDISRSGCSWTVGNGCFSAQQVKQMLLNRSTCPW
>Synspl
MEFPFDINQLFSERVSIIDHNLVGSPKSAGRSDLQSLIVTVVDELGRASAQAQNLPAPITSASKLQCQRHQLYLLKDGDANGGNGAILGY
LKIGSKKLFLLDRQGVHVEAEPECALDFYVAESMQRNGYGLELFNFMLQHKSLEPARMAYDRPSPKFLSFLAKHYCLNQSVPQVRFLQ
FFSTVTQLRKLVPKKPEGEIKPYSLTGREAVHQEQKMPPWPFTTTQSAVSQNCHSPSRTPRSIVVPPYGDNVDRNLGSPRIEHCGARRRS
>Synbia
MEFPFNINHLFSDRFSVLDNTFVASRVSQKRPDLQSHIATVIDEMGRASAKAQKLTTPVTSAAKLQSQHHQLYLMKDGESKGGCGAIVG
FLKVGYKKLFLLDQQGLHIEAEPLCVLDFFIAENSQRHGYGLELFNFMLQHKTSDPVQLAYDRPSPKLLSFLAKHYCLTQSVPQVNNFV
VFDGFFLNKSVEQSRMIPLKKPDTEIKPYSIMEREAVRQERRSLPWPFVPPHSPQRLVSSQFSLSLESSPTKSPLRASTLSAPGVHRDQSPN
FPLIERSRERRTSSQQGQVARDGLYSRYMDNKSLGMLGRPLPGLRSVGDRSQVLRRTETHRLVSVPILQSRLVTHPLPSATKDNVVCRP
TSLTNNRLQIGADAELAGVEKKLRPPNGTGNTARVLLESRRYLLARERDISRSGCCCIVGNTRFAAQQVKQMLLNRSTCPW

MOLECULAR ECOLOGY

>Synacu
MEFPFNVNQLFPERFSILDKSLVVEHASPKRADLQSHIATVIDELGRASAKAQKLTTPVTSATKLQSQHHELYLMKDGESNGECGAIVGF
LKVGYKKLILLDRRGLHTEAEPLCVLDFFIAENLQRHGYGLELFHFMLQHKNIDPVLMAYDRPSPKLLSFLAKHYCLTQSVPQVNNFVV
FEGFFNNKSVAKLRTIPLRKPDSEIKPYSLVEREAVRREERSLPWPLASSQCSLSVGSSPTRAALGLHRDQRPKFPLNEPSRERRTSQQGQ
VAREGLYSRHMDSRSLGRPLAGLRSAKEQAHLSQRLPKLQSRLRTPPTLSATEDNAVCWPTSPGRQKLRLGTDAEVADGKKELPAPNG
PGSAARVLSESPRHLSDGCPRTVGNACFSAQQMKQRLLLNRDTRPW
>Synros
MEFPFNVNQLFPERFSILDKSLVVEHASPKRADLQSHIATVVDKLGRASAKAQKLTTPVTSATKLQSQHHELYLMKDGESNGECGVIVG
FLKVGYKKLILLDRRGLHTEAEPLCVLDFFIAENLQRHGYGLELFHFMLQHKNIDPVLMAYDRPSPKLLSFLAKHYCLTQSVPQVNNFV
VFDGFFNDKSVARLRTIPLKKPDSEIKPYSMVEREAVRREERSLPWPLASSQCSLSVGSSPTRAALGVRRDQRPKLPLDEPSRERRTSQQ
GQVAREGLYSRHMDSRRPLAGLRSAKEQAHLWQRLPKLQSRLRTPPTLSATEDKTVCWPTSPGRQKLRLGTDAEVADGRKELPAPNG
PASAARVPSQSPRRLSDVCPRTVGKACFSAQQMKQRLLLNRDTRPW
>Synsco
MEFPFNVNQLFPERFSILDKSLVVEHASPKRADLQSHIATVIDELGRASAKAQKLTTPVTSATKLQSQHHEVYLMKDGESNGECGVIVG
FLKVGYKKLILLDRRGEHTEAEPLCVLDFFVAENLQRHGYGLELFHFMVQVNNFVVFDGFFNNKSVAKLRTIPLKKPDSEIKPYSIVERE
AVRREERSLPWPLASSQCSLSVGSSPTRAALGVRRDQRPNFPRNEPSRERRASQQGQVAREGLYSRHMDSRSLGRPLAGLRSAKEQAHL
SQRLPKLQSRLHTPPTLSATEDNALCWPTSPGRRKLRLGTDAEVADGEKELPAPNDEAALVVEQGHVSVVSRGPLSEDGLSKMEERCE
LQGTGALIMLLPALSFSEDDRNEQDVALLSALLQLKQLQRASTWHCPLPLVILVPGPDGITCNTGKLEEELMLPMLVKDGLISEYMLLFI
PESTNDMQGSKSLAALCVGLCPAHLHLSHFAAAPWRNSWRPT
>Syntyp
MEFPFNVNQLFPERFSILDKSLVVEHASPKRADLQSHIATVVDKLGRASAKAQKLTTPVTSATKLQSQHHELYLMKDGESNGECGVIVG
FLKVGYKKLILLDRRGLHTEAEPLCVLDFFIAENLQRHGYGLELFQFMLQHKNIDPVLMAYDRPSPKLLSFLAKHYCLTQSVPQVNNFV
VFDGFFNDKSVARLRTIPLKKPDSEIKPYSMVEREAVRREERSLPWPLASSQCSLSVGSSPTRAALGVRRDQRPKLPLDEPSRERRTSQQ
GQVAREGLYSRHMDSRRPLAGLRSAKEQAHLWQRLPKLQSRLRTPPTLSATEDKAVCWPTSPGRQKLRLGTDAEVADGRKELPAPNG
PASAARVPSQSPRRLSDGCPRTVGKACFSAQQMKQRLLLNRDTRPCCGVCLDPNHLKHFPL

>Antmac
MEFPFDINRLFSERVTVLDDSIIAGRKSAGRLICLCRPDLQAHISSVIDELGKASAM
AQQLPAPITSASKLQSQQHQLYLLKEGERNGGRGAIVGFLKVGHKKLFLLDLQGVHIEAE
PLCVLDFYISENLQRHGYGLELFDFMLQHKSLEPVLMAYDRPSPKFLSFLAKHYLLTKSV
PQVNNFVVFEGFFLNRSAAQL
>Antstr
MEFPFDINRLFSERVTVLDEGIIAGRKSAGRSICLCRPDLQAQISSVIDELGKASAM
AQQLPAPITSASKLQSQQHQLYLLKEGERNGGRGAIVGFLKVGHKKLFLLDLQGVHIEAE
PLCVLDFYISENLQRHGYGLELFDFMLQHKSLEPVLMAYDRPSPKFLSFLAKHYLLTKSV
PQVNNFVVFEGFFLNRSAAQL
>Loppis
MEFPSDINQLFSERISIMDHDLVAGRRTAGRKQMCVCLRRPDLQAQIATIIDALGRASAK
AQQLTAPITSASKLQSQKHQLYLMKDGESNGGRGVVVGFLKVGHKKLFLLDVHGVHVEAE
PLCVLDFYIAENVQRHGYGLELFDFMLQHKNLEPVLMAFDRPSPKLLSFLAKHYFLTQSV
PQVNNFVVFEEFFLNLSDSQL
>Acalat
MEFPFDINKLFSERVSVLDQTLIAGRSSAGRPDLQAHLTTVIDELGRASAK
AQQLTAPITSASKLQSQRHQLYLLKDGESNGGRGVVVGFLKVGYKKLFLLNPQGVHIEAE
PLCVLDFYIAENLQRHGYGLELFDFMLQHKNVEPVLMAYDRPSPKFLSFLARHYCLTQSV
PQVNNFVVFEGFFLNISAAQL
>Dipsar
MEFPFDINKLFSERVSILDQTLITSARRPDLQAHITTVIDELGRASSK
AQQLTAPITSASKLQSQRHQLYLLKDGESNGGRGVVVGFLKVGYKKLFLLNPQGIHIEAE
PLCVLDFYIAENLQRHGYGLELFDFMLQHKNVEPVLMAYDRPSPKFLSFLARHYCLTQSV
PQVNNFVVFEGFFLNISAAQL
>Spaaur
MEFPFDINKLFSERVSILDQTLIAGRTSAGRPDLQAHITTVIDELGRASAK
AQQLTAPITSASKLQSQRHQLYLLKDGESNGGRGVVVGFLKVGYKKLFLLNPQGVHIEAE
PLCVLDFYIAENLQRHGYGLELFDFMLQHKNVEPVLMAYDRPSPKFLSFLARHYCLTQSV
PQVNNFVVFEGFFLNISAAQL
>Datund
MEFPFDINQLFSERVSILDQNLVAGRKSAGRPDLQAHIATVIDELGRASAK
AQQLTAPITSASKLQSQRHQLYLLKDGESNGGRGVVVGFLKVGYKKLFLLDLQGVHVEAE
PLCVLDFYIAENLQRHGYGLELFDFVLKHKNLEPVLMAYDRPSPKFLSFLAKHYCLTQSV
PQVNNFVVFEGFFANRSAAQL
>Takfla
MEFPFDINQLFPERISVLDHTLEAGGKFAGRPDLQACCATVIDELGKASAR
AQQLPAPVTSASKMKSQRHQLYLLKDGESNGGRGVVTGFLKIGYKKLFLLDLQSVHVEAE

MOLECULAR ECOLOGY

```
PLCVLDFYIVESLQRHGYGLELFDFMLQDKNLDPVLMAYDRPSPKLLSFLANHYHLTENV
PQVNNFVVFKSFFLNRSGNIF
>Takrub
MEFPFDINQLFPERISVLDHTLEAGGKFAGRPDLQACCATVIDELGKASAR
AQQLPAPVTSASKMKSQRHQLYLLKDGESNGGRGVVTGFLKIGYKKLFLLDLQSVHVEAE
PLCVLDFYIVESLQRHGYGLEVFDFMLQDKNLDPVLMAYDRPSPKLLSFLANHYHLTENV
PQVNNFVVFKSFFLNRSGNIF
>Tetnig
MEFPFDIQQLFPGRVSILDHRPELQPLAAAVVDELGKASAR
AQQLPAPVTSASRLKSQRHQLYLLKDEDSNGGRGGVVGFLKIGHKKLFLLDPQSVHVEAE
PLCVLDFYIVENLQRHGYGLELFDFMLQDKNLDPVLMAYDRPSPKLLSFLAKHYRLTDSV
PQVNNFVVFRSFFLRRAG
>Thasep
MEFPFNINHLFPEKLSVLDCTLAAGPRSPRRPDHQASIATVIDELGKASAK
AQQLTAPITSASKLQSQRHELYLLKDAERNGGQGVIVGFIKIGYKKLFLLLLVGEHVEAE
PLCVLDFYIAENLQRHGYGLELFDFMLQHRTLDPVLMAYDRPSEKFLSFLAKHYDLTQSV
PQVVNNFVVFKSFFLNKSGSIF
```

Reference:

Yasumasu, Shigeki, Miyuki Horie, Mayuko Horie, Kodai Sakuma, Chihiro Sato, Hikari Sato, Taiki Nakajima, Tatsuki Nagasawa, Mari Kawaguchi, and Ichiro Iuchi. 2024. “Transglutaminase Mediates the Hardening of Fish Egg Envelope Produced by Duplication of Factor XIIIA Gene during the Evolution of Teleostei.” *Journal of Biochemistry* 176 (6): 427–36. https://doi.org/10.1093/jb/mvae062.
